# Pitfalls in Developing Machine Learning Models for Predicting Cardiovascular Diseases: Challenge and Solutions

**DOI:** 10.2196/47645

**Published:** 2024-07-26

**Authors:** Yu-Qing Cai, Da-Xin Gong, Li-Ying Tang, Yue Cai, Hui-Jun Li, Tian-Ci Jing, Mengchun Gong, Wei Hu, Zhen-Wei Zhang, Xingang Zhang, Guang-Wei Zhang

**Affiliations:** 1 The First Hospital of China Medical University Shenyang China; 2 Smart Hospital Management Department The First Hospital of China Medical University Shenyang China; 3 Shenyang Medical & Film Science and Technology Co, Ltd Shenyang China; 4 Digital Health China Co, Ltd Beijing China; 5 Bayi Orthopedic Hospital Chengdu China; 6 China Rongtong Medical & Healthcare Co, Ltd Chengdu China; 7 Department of Cardiology The First Hospital of China Medical University Shenyang China

**Keywords:** cardiovascular diseases, risk prediction models, machine learning, problem, solution

## Abstract

In recent years, there has been explosive development in artificial intelligence (AI), which has been widely applied in the health care field. As a typical AI technology, machine learning models have emerged with great potential in predicting cardiovascular diseases by leveraging large amounts of medical data for training and optimization, which are expected to play a crucial role in reducing the incidence and mortality rates of cardiovascular diseases. Although the field has become a research hot spot, there are still many pitfalls that researchers need to pay close attention to. These pitfalls may affect the predictive performance, credibility, reliability, and reproducibility of the studied models, ultimately reducing the value of the research and affecting the prospects for clinical application. Therefore, identifying and avoiding these pitfalls is a crucial task before implementing the research. However, there is currently a lack of a comprehensive summary on this topic. This viewpoint aims to analyze the existing problems in terms of data quality, data set characteristics, model design, and statistical methods, as well as clinical implications, and provide possible solutions to these problems, such as gathering objective data, improving training, repeating measurements, increasing sample size, preventing overfitting using statistical methods, using specific AI algorithms to address targeted issues, standardizing outcomes and evaluation criteria, and enhancing fairness and replicability, with the goal of offering reference and assistance to researchers, algorithm developers, policy makers, and clinical practitioners.

## Background

Cardiovascular diseases (CVDs) refer to both ischemic and hemorrhagic diseases that affect the heart, brain, and systemic vasculature, such as heart failure (HF), atrial fibrillation, acute coronary syndrome (ACS), myocardial infarction (MI), coronary heart disease (CHD), stroke, and cerebrovascular disease. As the most common noncommunicable diseases worldwide, CVDs remain a major cause of death in both low- and high-income countries. In 2019, there were 18.6 million deaths due to CVDs [1]. As awareness of the serious threat that CVDs pose to human health has grown, many studies have focused on developing tools and guidelines for predicting these diseases. These prediction models can help identify individuals at high risk of developing CVDs, enabling preventive measures to be taken in a timely manner and potentially reducing both the human and economic costs of the disease. In this context, effective CVD risk prediction and prevention are critical to addressing this global challenge [2,3]. As a result, developing reliable and feasible prediction models for CVDs has become an ongoing area of exploration and study.

Early in 1976, the first CVD risk prediction equations were developed by the Framingham Heart Study, called the Framingham risk score (FRS). As one of the most classic CVD risk models, this score was widely applied all over the world in the following decades to provide important guidance for public health and clinical practice [4]. With the development of CVD preventive research, other important prediction tools have emerged for regional applicability, such as the Systematic Coronary Risk Evaluation model in Europe, the QRISK in the United Kingdom, the pooled cohort equation (PCE) for atherosclerotic CVDs (ASCVD) reported recently by the American College of Cardiology and American Heart Association guideline, and so on [5,6]. These models have a common feature: they all consist of fixed equations and lack scalability and are thereby defined as traditional models.

Although traditional models remain the most popular tools in the field of CVD prevention, they have proven to be inefficient and inflexible in the face of rapidly expanding amounts and types of data and increasing clinical requirements for precise, comprehensive, and continuous CVD risk prediction and treatment recommendations. The ideal risk prediction models are expected to include the broadest possible range of parameters and clinically relevant outcomes and to provide real-time and continuous support for doctors’ decision-making, such as suggestions for smoking cessation, physical activity, diet, and medication use [7]. Obviously, the traditional models are no longer competent for these responsibilities.

Machine learning (ML) involves endowing computers with the ability to simulate or replicate human learning behavior, allowing them to acquire new knowledge or skills, reorganize existing knowledge structures, and continually improve their performance. As an important subset of artificial intelligence (AI), it has emerged as a promising research area in recent years [8-10]. ML models have also led to a significant evolution in the field of CVD risk prediction, allowing for the handling of new features of existing variables, such as nonlinearity and temporal dynamics, as well as novel variables such as electrocardiography results, medical images, and even genomics data [11-21]. Our recent systematic review [22], which included 486 AI-CVD prediction models across 79 articles, identified that AI has initiated a promising digital revolution in CVD risk prediction, characterized by an increase in the number and dimensions of predictors, as well as a notable diversity in applied algorithms, encompassing 66 specific algorithms across 13 categories.

Despite the stronger predictive ability and more promising development prospects of ML models compared with traditional ones, the development and clinical application of AI prediction technology are still strictly limited by a series of key problems. In the field of CVD prediction, this situation appears more severe than in other disease prediction or prognosis research [22-24]. As our systematic reviews have found [22], all current published AI-CVD prediction models exhibited a high risk of bias, lacked independent external validation, and had no clinical implementation. Moreover, the onset of CVDs is an exceptionally prolonged process, making prospective clinical validation of a flawed model potentially very costly in terms of research resources and possibly harmful to populations. Therefore, summarizing the potential pitfalls in AI-CVD prediction model research is crucial. Providing researchers with adequate warnings and references before initiating their studies is not only necessary but also of significant importance.

Although there are already some criteria in AI research that can be used as references, such as the Prediction Model Risk of Bias Assessment Tool (PROBAST) [25], Transparent Reporting of a Multivariable Prediction Model for Individual Prognosis Or Diagnosis (TRIPOD) [26], and AI transparent, replicable, ethical, and effective research (AI-TREE) [27], summarizing and analyzing key considerations and strategies during the research process will directly contribute to the practical implementation, replication, dissemination, and clinical application of model studies [28]. This holds significant practical significance not only for researchers but also for algorithm developers, cohort investigators, policy makers, health care providers, and professionals. Russo and Bonassi [29] previously outlined several “pitfalls” encountered in AI studies in nutritional epidemiology, including issues related to measurement methods, confounding factors, nonlinearities, missing data, overfitting, and interpretability, among others. Similarly, Chiarito et al [30] identified a few “pitfalls” in their review of AI and CVD risk prediction [30]. However, there is currently a lack of a comprehensive and systematic summary specific to the AI-CVD prediction field. In this paper [25,27,31], we summarize and analyze these existing problems and their possible solutions, aiming to provide guidance and references during the process of the development of AI models for the prediction of CVDs and other diseases.

## Design and Summary of a Framework for Presenting Existing Pitfalls

**Figure 1:**
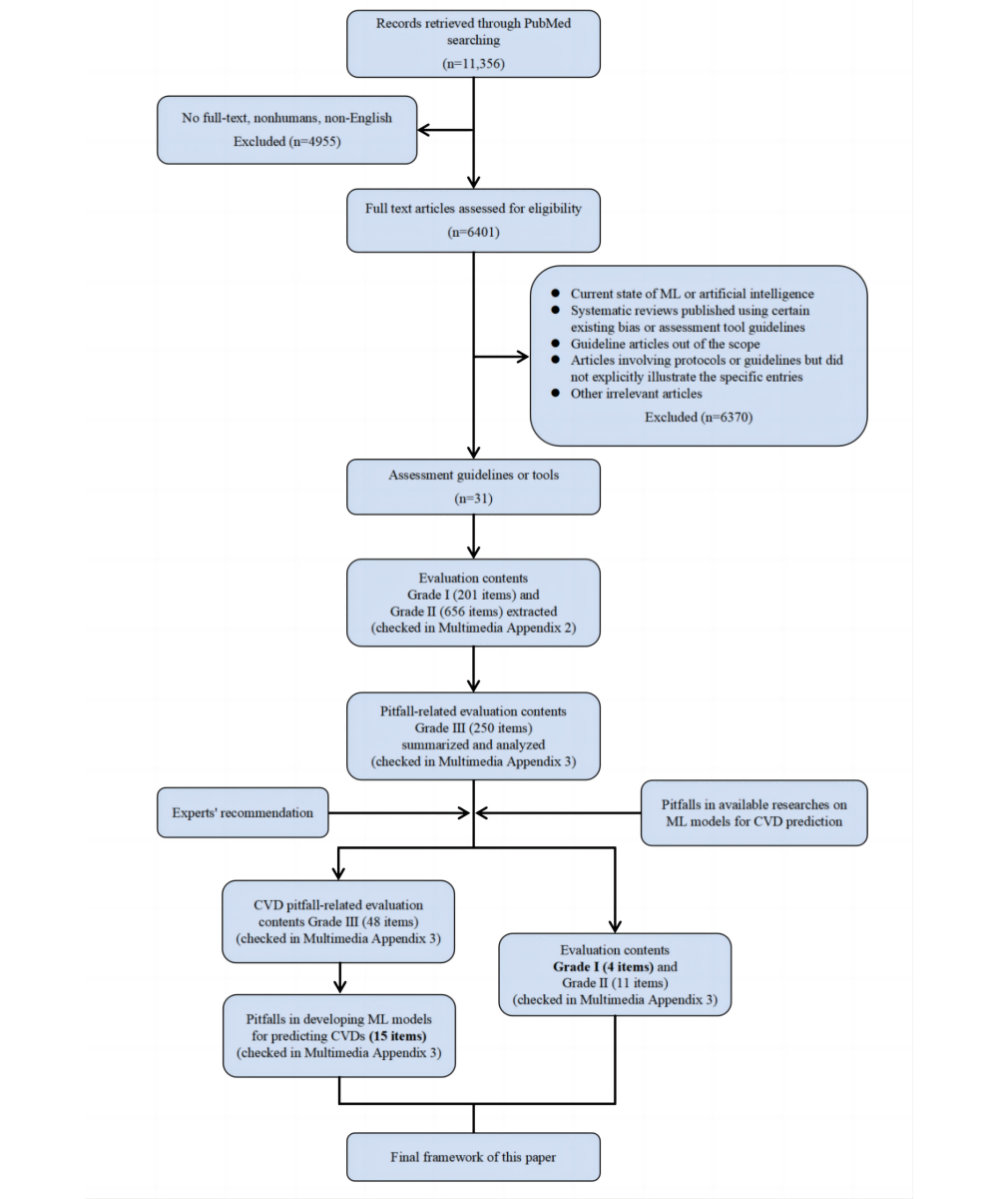
The flowchart for developing a framework for this paper based on the assessment guidelines or tools in the field of medical artificial or machine learning research. CVD: cardiovascular disease; ML: machine learning.

To develop a framework outlining the key pitfalls, the following process was executed (Figure 1). A literature review of existing assessment guidelines or tools related to AI or ML medical research was performed, with the search strategy of “assessment tool/guideline”+“AI/ML”+“bias” and their synonyms and related terms (details are shown in Multimedia Appendix 1). Full-text articles in English related to human participants were included, while low-quality or irrelevant articles, such as those discussing the current state of ML or AI or guidelines outside the scope of this study, were excluded. Detailed information is provided in Figure 1. The inclusion and exclusion process was conducted by YQC, YC, and GWZ. Ultimately, 31 papers were included in the preliminary summary of candidate items (Multimedia Appendix 2 [25,27,31-60]). Subsequently, incorporating the risk issues identified in our previous systematic review of AI-CVD prediction models [22], a final framework was further discussed and confirmed by a panel of experts. This panel included AI experts (TCJ and MG), statisticians (ZWZ), clinicians (DXG, XZ, and GWZ), and information technology specialists (WH), among others. As summarized in Multimedia Appendix 3 [15,18,21,28,61-100], the framework encompasses four major categories with 15 subcategories: (1) data quality (data source, subjective factors of researchers, incomplete data, and parameter acquisition methods); (2) data set characteristics (small sample size, low event rate, characteristics of the data distribution, and multimodal data); (3) model design and statistical methods (outcome definitions, incomplete inclusion of covariates, overfitting, and defects in evaluation criteria); and (4) clinical implications (generalization, interpretability, and AI ethics).

## The Effectiveness of the ML Models Still Largely Depends on Data Quality

### Overview

Data are undeniably a fundamental element in ML models, which are inherently sensitive to the quality of the data used for training and validation. The adage “garbage in, garbage out” is particularly pertinent here [101]; poor-quality input data can also result in a biased ML model, and even minor errors or biases in training data can lead to unforeseen consequences in a model’s predictions. These considerations raise ethical concerns regarding the reliability of decisions based on ML model predictions. Consequently, understanding and mitigating pitfalls in data collection, preprocessing, analysis, and application become critically important. Specific methods involve selecting objective indicators collected through reasonable means based on a thorough evaluation of data authenticity.

### Data Recourse Is the Key Factor

It is well-known that data accuracy is a key influencing factor for data-based prediction models, as inaccurate data can significantly impact the predictive ability of subsequent models. One of the main reasons for data inaccuracy is the collection of subjective data, such as self-reported data on blood pressure (BP), obesity, and family history, as noted by Hippisley-Cox et al [102] and Manuel et al [103] in traditional models, based on the Canadian Community Health Survey (104,219 participants) and QResearch database (3,610,918 participants), respectively. In fact, AI models have encountered the same dilemma [104,105]. As reported by Han et al [61] in their ML research based on the Korea Initiatives on Coronary Calcification (86,155 patients), inaccurate data from self-administered questionnaires had a limited contribution to model development; after feature selection based on information gain ranking (such as the history of diabetes and hypertension, this limitation resulted in an exclusion of important predictive factors from 10 most pertinent variables of a future all-cause mortality (ACM) events. Ultimately, this underestimation of important risk factors may lead to insufficient attention being paid to them, thereby misdirecting primary prevention strategies such as aggressive glycemic control.

Similar to traditional models, the most effective solution in AI models is still to collect objective data, instead of relying solely on self-reported data whenever possible. Even electronic health records (EHR) can contain incorrect data, which may be difficult to detect and therefore easily overlooked [62]. For example, in a study conducted by Rodriguez et al [63], race data, which were obtained from a community-based EHR with 231,622 participants, were mainly self-reported, while other data were inferred through validation methods that were based on PCE, leading to possible misclassification, and may affect the risk prediction for ASCVD. By way of illustration, it is advocated by Teoh [64] that the diagnosis or the value of the check index should be applied to further confirm the disease status of the participants, including hypertension, diabetes, and dyslipidemia reported by the participants themselves, and they found that the area under the curve (AUC) dropped from 0.623 to 0.608 when the diabetes examination inputs were removed (including blood glucose, glycated hemoglobin [as standardized by Japan Diabetes Society], and glycated hemoglobin [as standardized by National Glycohemoglobin Standardization Program]). These examples clearly demonstrate the impact of data authenticity on the robustness of the models. Unfortunately, even some classic scoring tools, such as PROBAST, TRIPOD, and CREMLS (Consolidated Reporting Guidelines for Prognostic and Diagnostic Machine Learning Models), do not specify methods for collecting objective data [32,33,106,107]. Therefore, we suggest the implementation of appropriate validation measures during model development.

### The Subjective Factor of Researchers Is a Potential Interference

In some studies, basic background, sociodemographic, and follow-up information may be collected by local doctors and nurses [65,108,109]. Although these interviewers have been strictly trained, the data collection process is likely to be interfered with by their personal thoughts. Benjamins et al [66] reported that in the Glycometabolic Intervention as Adjunct to Primary Percutaneous Intervention in ST-Segment Elevation Myocardial Infarction-III study, which included 222 people and defined ventricular function dysfunction as an outcome, using data created by local observers to train and validate the U-Net model may result in an unstable model quality. Liu et al [67] analyzed the registry data of 53,213 inpatients in the Cardiovascular Department of Xiangya Hospital; they found that student extractors, who manually filled missing data using a hierarchical mean filling method, subjectively adjusted variable parameters of the extracted data based on experience and introduced a potential risk of bias. Although these reports mention the potential impact of researchers’ subjective factors on the models, they lack effective proof methods, highlighting that such factors are often overlooked and difficult to evaluate. Therefore, it is essential to identify subjective factors of researchers before the study.

The most common and effective approaches to address this issue are to improve training and supervision of data collectors. While both traditional and ML models require manual data processing, some studies have shown that AI can potentially replace humans in data processing to reduce the impact of subjective factors. For instance, in the Systolic Blood Pressure Intervention Trial (SPRINT) and the Hong Kong eHealth cohort studies with 8133 and 1094 participants, respectively, Tsoi et al [68] used a K-means clustering ML algorithm to identify variations in BP without the need for human adjustment, which resulted in low clustering similarities (Davies-Bouldin Index: 0.653 in SPRINT and 0.680 in the Hong Kong eHealth cohort). This not only further highlights the importance of controlling subjective factors in model construction but also provides an alternative solution beyond simply enhancing training.

### Incomplete Data Also Affect the Effectiveness of ML Models

Incomplete data are a common problem in retrospective studies and may significantly impact the final predictive results of ML studies. For instance, in the Melbourne Collaborative Cohort Study with 32,611 participants, Sajeev et al [69] reported that 32% of high-density lipoprotein cholesterol data were missing and that this resulted in a lower AUC of 0.753 (95% CI 0.729-0.777) in a logistic regression (LR) model, while an AUC of 0.874 (95% CI 0.833-0.915) was achieved in another similar LR model that was developed by the North West Adelaide Health Study, which had only 41 missing data among 3654 participants. It is also argued by Han et al [61] that unmeasured factors could introduce bias in their ACM prediction ML model, which used the Korean Initiatives on Coronary Calcification registry, a retrospective cohort. The severity of this issue lies in its potential to also cause underestimation or neglect of the impact of risk factors in actual clinical practice, thereby affecting CVD prevention strategies.

Both traditional and ML models face the issue of missing data. In traditional models, missing data are often directly excluded, which can impact model stability and weaken its significance [70,108,110]. In contrast, ML models use several statistical methods to address the problem of missing data and optimize the models, such as multiple imputation, multiple imputation with chained equations, logitBoost, multiple imputation by fully conditional specification, Markov chain Monte Carlo method, median imputation, and k-nearest neighbor (KNN) imputation [71,72,111,112]. The application of these statistical methods gives ML models an advantage over traditional models in handling missing data. For instance, in a study that included 423,604 participants from the UK Biobank, Alaa et al [73] used the missForest algorithm to impute missing data in an ML model, achieving a significantly higher AUC of 0.774 (95% CI 0.768-0.780) than with FRS (AUC=0.724, 95% CI 0.720-0.728). In the ATTICA prospective study involving 2020 participants, Dimopoulos et al [112] excluded variables with missing values in >70% of the individuals. In order to improve the performance of the CVD risk estimator, the missing data in part of the remaining 22 variables were then imputed by KNN imputation, resulting in relatively high accuracy rates (KNN: 96%, random forest [RF]: 99%, decision tree classifier: 99%). Therefore, ML models can better manage missing data due to the application of the aforementioned statistical methods. Furthermore, ML models also can register patients with missing necessary variables in traditional models [62], providing an advantage in handling incomplete data. In addition, besides the aforementioned statistical methods, Weng et al [71] used a novel approach in the Clinical Practice Research Datalink (CPRD) study that included 378,256 patients, using missing data as an independent variable to predict the risk of CVDs. Specifically, they created dummy variables to indicate missing continuous variables and a separate category of “unknown” to represent missing categorical variables such as the Townsend deprivation index and race, with an acceptable AUC of 0.764 (95% CI 0.759-0.769) and a specificity of 70.7% in the neural networks model eventually. Although this method needs to be further confirmed, exploring such new approaches provides additional options for ML models in handling missing data, thereby enhancing AI-CVD prediction models.

### Appropriate Parameter Acquisition Method Is Another Important Factor

In most studies predicting the risk of CVDs, researchers have typically used one-time measurements of risk factors, ignoring the fact that some factors exhibit significant variability over time, such as systolic BP (SBP), plasma glucose levels, low-density lipoprotein, and serum total cholesterol [113-115]. As demonstrated in the SPRINT study mentioned in The Subjective Factor of Researchers Is a Potential Interference section, BP measured at fixed times (visit-to-visit BP variability) can vary greatly from 24-hour ambulatory BP variability (*r*^2^<0.026), for the data that are acquired intermittently with a short follow-up time may introduce bias in the data [68]. These common problems illustrate that variables measured at a certain time point cannot necessarily represent the true level of the variable in the population. In fact, there have already been diagnostic criteria for multiple measurements of some risk factors in guidelines, for single measurements are likely to introduce significant bias within clinical work. For example, Stergiou et al [116] advocated that a minimum of 2 to 3 office visits at 1- to 4-week intervals are frequently necessary for the office BP assessment.

The solving method is to use repeated measurements of risk factors to improve the model prediction [117]. In the National Health Insurance System-National Health Screening Cohort (NHIS-HEALS) study with 361,239 participants selected, Sung et al [74] advocated that by offering discrimination and calibration with repeatedly measured data, the deep learning model takes advantages in CVD risk prediction in the EHR era (female participants: AUC=0.94, 95% CI 0.91-0.97 in; male participants: AUC=0.96, 95% CI 0.95-0.97 in). In another study including 80,964 people, multiple SBP recordings from EHR (provided by UK primary care) were analyzed. It was found that the multiple SBP recordings had a better correlation with CVDs than a single recording, as identified by an increased hazard ratio from 1.22 (95% CI 1.18-1.30) to 1.39 (95% CI 1.31-1.46) with the use of repeated measured factors [75]. Besides, in the NHIS-HEALS study, Cho et al [76] handled continuous variables by using their mean, minimum and maximum values, and SDs in the developing of recurrent neural network models and achieved a high level of discriminative accuracy (female participants: AUC=0.921, 95% CI 0.908-0.934; male participants: AUC=0.896, 95% CI 0.886-0.907), which was highlighted by authors as significant improvement compared with single-measured method [76]. Furthermore, collecting data multiple times can enhance data consistency and to the benefit of exploring the changing trend of data.

## Data Set Characteristics Largely Affect the Effectiveness of the ML Models

### Overview

The impact of the data set characteristics used for ML on risk prediction is crucial and should not be disregarded. This includes several elements: the impact of the small sample size and low event rates, the alignment of data distribution characteristics with the model, and the challenges associated with the application of multimodal data. These pitfalls can significantly impede ML studies, highlighting the critical need for heightened attention in the various stages such as research design, implementation, data analysis, model construction, paper preparing, and even in submission and peer review processes. Specific methods include using sufficiently large cohorts, extending follow-up periods, using specific AI algorithms to address targeted issues, and selecting clinical routine examinations as risk factors whenever possible.

### Small Sample Size Is a More Serious Problem

It is well known that insufficient sample size will lead to the risk of bias for the study in the traditional model, and the evaluation standard of sample size has been developed by the PROBAST [106]. Therefore, in order to improve the credibility of research conclusions, more and more researchers have used large data sets that are multiethnic cohorts with high quality [77,118-120], although there are still many substandard studies [108,121]. However, in the field of ML prediction models, the problem of insufficient sample size seems to be more serious. In our previous research of 79 CVD risk prediction studies, the number of participants included in the ML model could even be as low as 80 [22]. The lack of a sufficient sample has become the biggest contributor to the high risk of bias [28]. For example, in a retrospective observational study of 420 patients, Ponomartseva et al [78] stated that when the sample size is smaller than the optimal ML method size, the accuracy of the model may change. In another study carried out with 451 consecutive patients from a tertiary hospital, conventional univariate and multivariate analyses were limited because of the small sample size; the median accuracy of artificial neural network (ANN) models in predicting recurrent stroke was only 75%, as reported by Chan et al [79].

Although a larger population size was prone to the better performance and reliability of the models, as revealed by Alaa et al [73] in an experiment with a series of subpopulations of varying sizes and a fixed number of variables, the sample size of the ML algorithms is dependent on various factors, including the types of algorithms, the number of index, the features of the sample, and so on [122-124]. For example, the classical events per variable method, which often follows the “one in ten rule” for the sample size calculation, may not be applicable to certain ML algorithms due to their specific operation mechanism [22]. Therefore, it is strongly recommended that the sample size calculation be performed before initiating ML research, considering the various factors mentioned above, due to dramatic differences in the mechanisms of sample size calculation between ML and traditional models. Meanwhile, the upcoming PROBAST-AI, a special tool for bias evaluation for AI models, has been expected to provide a reference standard [125-127]. Before this, we are inclined to recommend using the “one in ten rule” as the minimum standard to include as many samples as possible.

### Application of the ML Models Is Also Troubled by Low Event Rate

The low incidence rate, a common problem for prediction model construction, may restrict the generalizability and predictive ability of the prediction model in practice [74,80,128]. It may lead to the bad performance of the model in general or cause bias in predictions for a particular segment that has a low incidence rate. For example, on the basis of the data from 13,291 participants from 7 epidemiological cohorts, Jdanov et al [77] found that the low incidence rate of CVD deaths (<7% in male participants and <1% in female participants) at a young age (aged 45-54 years) affected the prediction outcomes at young ages, with a substantial underestimation of CVD mortality risk by 40% to 45% for male individuals and 3% to 4% for female individuals. Therefore, based on this reasoning, if applied in clinical practice, the low event rate may result in the underestimation of a substantial number of individuals who are at high risk for CVD, thus reducing the role of primary prevention.

The problem of low event rate can be overcome by the following methods, such as filtering of oversampled data using noncooperative game theory [80], synthetic minority oversampling technique [129], random oversampling [130], random undersampling [19], bootstrapping [81], and the stacking paradigm [131]. However, these methods can only improve the performance of the ML from the statistical method and cannot solve the problems with the data themselves. By using the data solely from the DaVita Inc EHRs (124,097 participants), Goldstein et al [82] reported a direct way to increase the incidence rate by broadening the outcome range and showed good training and test set loss at the same time. However, by using this method, the outcome will become more heterogeneous and will decrease the quality of the prediction model, and thus this method may not be an adequate option. The better approach may be to extend the follow-up duration or to include more different populations as mentioned above to improve the reliability of ML [61]**.**

### Characteristics of the Data Distribution Underscore the Critical Importance of Model Selection

The cornerstone of constructing predictive models lies in the analysis, mining, and use of data relationships between covariates (risk factors) and gold standards (outcome events). Traditional statistical methods often rely on the assumption of linearity when building predictive models. However, it is essential to acknowledge that in clinical practice, many risk factors and outcome events exhibit nonlinear relationships, although linear relationships may predominantly characterize some factors, such as glycated hemoglobin or smoking pack-years. For instance, Angeli et al [83] and Lip et al [84] reported a J-curve relationship between BP and CVDs or ACM. Similarly, dietary factors such as salt, carbohydrates, and fats have demonstrated U- or J-shaped relationships with CVD outcomes [132,133]. Unfortunately, certain ML algorithms are not well suited to handle nonlinear relationships, including linear regression, LR, and so on. Consequently, if linear algorithms are used to construct predictive models that incorporate nonlinear elements, the result may lead to inaccurate associations and distorted effect estimates. In a study based on the National Health and Nutrition Examination Survey cohort (37,079 participants), Dutta et al [85] developed 6 AI models with nonlinear factors (eg, BP) for predicting CHD, and expectedly, the LR algorithm achieved the worst performance (AUC=0.713). Similarly, Wang et al [70] included 40,711 participants from the Life Risk Pooling Project cohort to build 4 AI models with nonlinear factors (eg, BP) for predicting CVDs and also found the worst performance in linear naive Bayes algorithm (AUC=0.786, 95% CI 0.726-0.735; *P*=.001) [70]. In addition, many predictive models aimed to assess long-term risk for adverse events, but commonly used ML models often struggle with handling time-to-event variations and censored patient data, significantly limiting their performance. Li et al [72] demonstrated that LR algorithms that neglect censoring tend to substantially underestimate CVD risk to only 2.2% to 5.8% when compared with Cox models that had a risk of 9.5% to 10.5%, which are better suited for survival data analysis.

Therefore, it is imperative to emphasize that the application of nonlinear AI algorithms plays a pivotal role in the research of constructing predictive models that encompass intricate data relationships. These algorithms have the capability to capture and model intricate patterns, interactions, and dependencies within the data that may not be adequately addressed by linear approaches. As in the 2 examples elaborated in the previous paragraph, support vector machine (SVM) had the best performance with an AUC of 0.776 in the study by Dutta et al [85], and RF had the best performance with an AUC of 0.892 in the study by Wang et al [70]. When it comes to survival data, random survival forest (RSF) appears to exhibit superior performance, owing to their ability to handle complex, time-dependent relationships and censored data more effectively, ultimately resulting in more accurate survival predictions. For instance, in a study based on the Atherosclerosis Risk in Communities cohort (14,842 participants), Zhuang et al [86] developed 4 survival models (Cox proportional hazards model, Akaike information criteria for Cox regression, least absolute shrinkage and selection operator for Cox regression, and RSF), and the RSF algorithm achieved the best performance in predicting CHD (AUC=0.80, 95% CI 0.79-0.81) and ACM (AUC=0.78, 95% CI 0.77-0.78). Another example is meant by Ambale-Venkatesh et al [87], where 6814 participants from the Multi-Ethnic Study of Atherosclerosis (MESA) cohort were used for ACM risk. Eventually, the RSF algorithm (AUC=0.84) performed the best, yielding better results compared to those obtained with other algorithms (eg, Cox least absolute shrinkage and selection operator: AUC=0.80).

### Multimodal Data–Based Prediction Model Pitfalls Limit Clinical Applications

The number of studies of multimodal data–based prediction models for CVDs has risen substantially recently, and there have been a variety of data types, such as radiomics [88,134], proteomics [135], and genomics [136]. The multimodal data–based prediction model, which integrates available heterogeneous data into a unified framework, can fully consider the importance of each modality and incorporate information from multiple aspects, thus improving model performance [136]. However, the clinical accessibility of the used multimodal data presents another pitfall. As advocated by Pujadas et al [88], there was a limitation in the multimodal data–based CVD prediction model with cardiovascular magnetic resonance radiomics, because cardiovascular magnetic resonance was not a routine examination. More significantly, according to the AI-TREE criteria, the inclusion of unconventional or difficult-to-obtain examination results is a crucial factor influencing the clinical application of the model [27]. This may be solved by applying more commonly used tests (eg, electrocardiogram [ECG]) or disease-specific imaging examinations. For instance, it is reported by Lou et al [89] that ECG-based patient characteristics from 2 hospitals in the Tri-Service General System were used for CVD risk prediction, and the deep learning models performed well, yielding excellent results (AUC>0.90). Another example of the solution was carried out by Chao et al [21], who included clinical routine 30,286 low-dose computed tomography data from 2085 patients with lung cancer in the National Lung Cancer Screening Trial to construct a deep learning CVD risk prediction model. Therefore, we suggest that multimodal model studies should focus on the practicality of data acquisition to enhance the feasibility of primary prevention of CVDs.

In addition, studies emphasizing multimodal data often disregard the inclusion of classical risk factors, neglecting essential parameters crucial for comprehensive prediction models. The overarching focus on multimodal data variables can lead to decreased model performance due to the absence of integrated incorporation of traditional risk factors. For instance, in the ECG methods, for the prompt identification of coronary events study (499 patients), Al-Zaiti et al [15] constructed ACS risk prediction models by using 554 temporal-spatial features of the 12-lead ECG without clinical examination factors such as total cholesterol or lifestyle variables such as smoking, which led to unsatisfactory validation performance (LR model: AUC=0.67; gradient boosting machine [GBM] model: ACS=0.71). Therefore, it is strongly recommended that the inclusion of classic predictive factors should form the cornerstone for constructing multimodal models. This is evident in the Singapore epidemiology of eye diseases study (comprised of over 70,000 images), where Cheung et al [134] not only applied imaging data but also comprehensively incorporated multiple types of variables in CVD risk estimators, and the best model achieved a perfect AUC of 0.948 (95% CI 0.941-0.954).

## Challenges Arising From Model Design and Statistical Methods

### Overview

In AI-CVD prediction research, model design and statistical methods play a critical role not only in aspects of model performance such as discrimination and accuracy but also in reproducibility and generalizability for clinical application. Key pitfalls in this area include issues of outcome definitions, incomplete inclusion of covariates, overfitting, and inadequate evaluation metrics. Some of these pitfalls stem from inherent problems associated with AI algorithms, necessitating researchers to thoroughly understand and mitigate their impact. In addition, other pitfalls can arise from design and implementation choices made by researchers, underscoring the need for careful planning and execution to avoid such issues during the research process. Specific methods include designing studies with standardized outcomes and evaluation criteria, incorporating all necessary variables, and using appropriate algorithms and validation techniques.

### The Outcome Definitions May Be Detrimental to the Significance of the Study

The definitions of the CVD outcomes show considerable heterogeneity in both traditional and ML models, concluding from the facts that there have always been some differences more or less in the detailed definitions among almost all studies. All the 3 versions of QRISK set the end points as CVDs, which are defined as a composite outcome of CHD, stroke, and transient ischemic attacks [5,111,137]. Systematic Coronary Risk Evaluation in Europe aimed to predict fatal CVDs, so its end point was CVD mortality [138]. PCE and Prediction for ASCVD Risk in China defined ASCVD as nonfatal MI, CHD death, or fatal or nonfatal stroke [139,140]. The outcomes of adding social deprivation and family history to CVD risk assessments were CVD-cause mortality, CHD, cerebrovascular disease, or interventions for coronary artery (coronary artery bypass grafting or percutaneous transluminal coronary angioplasty) [141]. These differences, to some extent, limit the comparability, reliability, and generalizability of the models, thereby affecting their clinical applicability for CVD prediction.

In studies of ML prediction models, heterogeneity is even more significant, with various origins of outcome definitions, such as disease codes (*International Classification of Diseases [ICD]*, *Ninth Revision* [*ICD-9*] or *ICD-10*), self-reports, and other international guidelines. For example, in a study with 31,466 participants from the UK Biobank, You et al [90] defined CVD events as MI or stroke with *ICD-9* and *ICD-10* codes. Conversely, Cho et al [76] in the NHIS-HEALS study, developed risk prediction models that used death from CVDs, MI, coronary arterial intervention, or bypass surgery stroke as the outcome definitions with only *ICD-10* codes. This heterogeneity in outcome definitions can lead to significant issues such as bias in model performance evaluation, reduced generalizability, challenges in data integration and standardization, difficulties in clinical application, and even misguidance in CVD prevention strategies. Therefore, it is crucial to address this problem adequately. To mitigate these issues, we strongly recommend that risk prediction models be developed with standardized use of *ICD* codes. This standardization would help in achieving consistent outcome definitions, thereby improving the reliability, generalizability, and clinical applicability of the prediction models.

### Incomplete Inclusion of Covariates May Diminish the Study’s Value

In many CVD risk prediction studies, several classical risk factors have been omitted. This phenomenon is not limited to retrospective studies using existing databases but is also prevalent in prospective research. By way of illustration, Chua et al [81] included 638 participants from Sandwell and West Birmingham Hospitals NHS Trust (local data set) to build 2 atrial fibrillation risk calculators. They did not include classical factors such as BP and smoking status and achieved a low accuracy performance with an AUC of 0.684 (95% CI 0.62-0.75) and 0.697 (95% CI 0.63-0.76), respectively. In the China Health and Retirement Longitudinal Study, Chen et al [91] included 9821 participants in the development of a stroke risk calculator. However, this classical public data set lacked classic variables such as total cholesterol, which resulted in a mediocre performance (AUC=0.7388) in the model. Such omissions may potentially lead to inadequate consideration of confounding factors, resulting in model bias and decreased performance or even rendering the model practically inapplicable [142]. From a statistical perspective, these classic risk factors can be considered confounders, and ignoring them may cause confounding, which represents a critical error and is a primary source of systematic errors that can manifest when assessing causality, potentially leading to misinterpretation of the results [143]. In accordance with the AI-TREE criterion [27], a key consideration is whether the available data can effectively address the clinical question at hand. In other words, a data set lacking essential predictors that are known to be relevant to an outcome is unlikely to satisfactorily address related inquiries. Therefore, studies that exclusively focus on model development while disregarding considerations for clinical application may deviate from sound research principles.

One potential approach to mitigate these challenges is to ensure that potentially biased features, such as ethnicity and social determinants of health, are explicitly incorporated into the models [142,144]. As suggested by Suri et al [28] in a study on understanding bias in ML systems for CVD risk assessment, achieving a robust ML-based design for CHD and CVD prediction necessitates the integration of traditional, laboratory, image-based, and medication-based biomarkers. In the realm of clinical research, where there is often a high number of variables and confounding factors, the adoption of high-capacity AI models is warranted [145,146]. For instance, Kakadiaris et al [80] obtained 6459 participants from the MESA cohort to build a CVD risk calculator based on SVM. They included all classic risk factors such as age, sex, smoking status, diabetes, SBP, total cholesterol, and high-density lipoprotein and achieved a rather high AUC of 0.94 (95% CI 0.93-0.95). In addition, in a study conducted with an outpatient health care system, Ward et al [62] enrolled 262,923 participants with risk factors that contained all classic risk factors as mentioned above for ASCVD risk prediction, and the AI model performed well with an AUC of 0.835 (95% CI 0.825-0.846). These also indicated the importance of incorporating complete covariates for accurate modeling, which is more conducive to individualized, precise, systematic, and comprehensive prediction and assessment of CVDs. This, in turn, facilitates targeted prevention and monitoring.

### Overfitting Determines the Performance of ML Models

Overfitting, a more severe problem in ML, means that the data can fit relatively perfectly with the derivation queue during training and derivation, but it will yield large biases and unreliable results when applied to other data, consequently leading to difficult generalization of the model [147,148]. The influence factors accounting for overfitting of ML models are summarized as follows: the features of algorithms, the number of variables, cohorts, outcome events, and so on. It is found in the research performed by Weng et al [71] that the more complex the model, the more likely it was prone to overfitting, while Commandeur et al [92] revealed that the few numbers of the derivation or the low event rate will cause the overfitting problem, which can be assessed by comparing the accuracy of the training set and the test set. These reports suggest that selecting appropriate algorithms and addressing the issue of low event rates have a decisive impact on preventing model overfitting, necessitating thorough consideration by researchers during study design.

There are numerous approaches to reduce the impact of overfitting, and the most commonly used include pretraining, hyperparameter selection, regularization, and cross-validation [149,150]. There are also other approaches, such as data-smoothing techniques, separating training, tuning, testing data, and the filtering of oversampled data using noncooperative game theory algorithm [80,92]. In addition, in the Prospective Cardiovascular Münster Study, which included 5159 participants, Voss et al [93] have proposed 3 solutions to address overfitting for CHD event estimations: cross-validation, stopping training when the errors in validation data sets are at a minimum, and rendering the networks with synthetic data and modifying them until the results are plausible. Eventually, the average negative log likelihood (ANLL) that explains the loss function of the model and the bias value attained comparatively minor numbers in the probabilistic neural networks model for both training (ANLL: 0.1712, bias: 0.0396) and testing sets (ANLL: 0.1807, bias: 0.0396).

### Inadequate Evaluation Metrics Still Exist in the Field of ML Model Prediction

The evaluation metrics help researchers assess the performance of models, and thus, defects in them can lead to obstacles in drawing practical conclusions. For example, although the AUC is the most commonly used evaluation criterion for model assessment, it may not accurately reflect the risk of CVDs in the population. This is because a person with a very low risk makes the same contribution to the AUC’s value as a person with a very high risk, as noted in a study developed by the CPRD with 3,660,000 participants [72]. Similarly, the Net Reclassification Index has been shown to be insensitive to changes in the model. For example, in a MESA study (5878 participants) reported by Polonsky et al [94], the Net Reclassification Index remained substantially constant even after including or excluding certain participants. These examples indicate that model evaluation cannot be adequately represented by a single metric; rather, it should be a comprehensive, multifaceted, and complex system, especially for AI models, in line with preventive medicine principles.

It is advisable to use a comprehensive evaluation of predictive model capabilities by considering various metrics such as accuracy, sensitivity, precision, specificity, *F*_1_-score, decision curves, and so on [151], which are also required to be reported in ML prediction studies as advocated by several reporting guidelines [32-34]. This is not only crucial for assessing the reliability of the model but also beneficial for its accurate application. Some commercial medical devices, such as ultrasound-assisted ECGs, have already contained criteria with high transparency and high clarity that are not available for predictive models. Thus, marketizing prediction models may be a solution. Moreover, in contrast to the predictive model, the intervention AI evaluation has different criteria, which are more rigorous and systematic. For instance, as the guideline of clinical trial reports for interventions involving AI, CONSORT (Consolidated Standards of Reporting Trials)-AI recommended presenting absolute and relative effect sizes for binary outcomes [35]. Therefore, we recommend that during model development, comprehensive calculation of these evaluation metrics is essential, as it is crucial for subsequent model dissemination and selection for clinical applications.

## Clinical Implication Remains a Significant Challenge in This Field

### Overview

In fact, the factors affecting the clinical application of AI-CVD models are complex. Beyond the pitfalls discussed above, 3 directly related and most prevalent factors that researchers should particularly consider are the problem of generalization, the lack of interpretability, and the limitations of AI ethics. These factors not only require researchers to minimize their impact during the study process but also necessitate collaborative efforts from clinicians, policy makers, and even the general public to overcome and address them. This includes clinicians integrating AI tools into their practice, policy makers establishing supportive regulatory frameworks, and the general public engaging in discussions about the ethical implications.

### The Problem of Generalization Has Largely Limited ML Algorithms Application

Generalization, which represents the ability to adapt to novel situations, is rather important for the applicability of CVD risk prediction models. However, both traditional and ML models have the problem of generalization, as they are developed based on specific populations. For example, many studies have used cohorts predominantly consisting of White individuals, men, or participants from a single center, even during a relatively narrow socioeconomic range with well-educated and low-risk factor burden compared with the general population [152-155]. Thus, risk prediction models were largely restricted to application of cross-cohorts. The Pooled Cohort Equations to Prevent Heart Failure tool, which was developed with the data of 11,771 individuals from 7 community-based cohorts, was unable to offer precise HF risk estimates for individuals who were not classified to the non-Hispanic White and non-Hispanic Black racial or ethnic groups [7]. The Cox regression models developed by Li et al [156] contained data from 44,869 participants from the Evidence for Cardiovascular Prevention from Observational Cohorts in Japan research group. Models were developed to predict death from CHD, stroke, and CVDs, but they may lack accuracy when predicting risk in women, for the corresponding *P* values of the Hosmer-Lemeshow test were .27, .002, and .04 in women and .51, .49, and .25 in men, respectively.

In ML, it is still a challenge to generalize models with specific populations, although this problem has been improved to a certain extent due to the elimination of some restrictions by AI algorithms. It has been documented by Bouzid et al [18] that using patients from only 1 region (even multiple hospitals) would significantly influence the promotion of ML models. In terms of improvement, it has been proven by Ward et al [62] that ML models developed in the United States or Europe could be expanded to Asian and Hispanic populations, and they achieved AUCs of 0.803 (95% CI 0.743-0.863) and 0.768 (95% CI 0.663-0.874), respectively, without the limitation of race in the PCE. In addition, the application of PCE is limited to the population aged from 40 to 79 years, while ML models have eliminated this limitation [62]. Moreover, the LR and SVM algorithms have been confirmed to overcome the age limitation (<79 years), which caused 26% of all cases in FRS not to be classified [95].

The way to solve the problem of generalization is to include more participants in a large data set to improve the models’ applicability as advocated by Chang et al [157] and Chen et al [158]. For example, it has been demonstrated that the model trained on the largest training set yielded the best performance in external validation [11,159]. Zarkogianni et al [96] have introduced larger data sets corresponding to patients with different ethnicities and races to other cohorts of patients and consequently extended the applicability of the model. Therefore, it is advocated to conduct multicohort, multicountry, or multiracial studies with adequate cross-validation. In the real world, however, it is difficult to obtain large data with high quality, which will cost lots of manpower and money and take a long time. In response to this point, it may be a more appropriate solution to fully use open-source databases, such as the National Health Insurance Service-National Sample Cohort, NHIS-HEALS, Qresearch, Early Identification of Subclinical Atherosclerosis by Noninvasive Imaging Research, and so on [76,92].

### The Lack of Interpretability in ML Algorithms Poses a Significant Challenge

Prediction models play a crucial role in helping doctors make accurate decisions in clinical practice. However, the “black box” effect in many ML algorithms, such as ANNs and RF, creates a challenge for doctors to interpret mechanisms correctly [160]. This limits the clinical utility of the models, as they become complex for doctors to understand the impact of risk factors on the prevalence of CVDs. In contrast to the fixed regression coefficient in traditional models, the complexity of ML algorithms hinders their interpretability, posing a common problem for doctors [95,160].

To overcome or reduce the negative impact of the “black box,” Han et al [61] suggested that information gain ranking methods could be used, while Weng et al [71] advocated for the use of data visualization to facilitate interpretation. Besides, in the ECG methods for the prompt identification of coronary events study with 1244 participants, Al-Zaiti et al [15] revealed that by incorporating feature selection and annotation based on clinical knowledge, LR could achieve comparable performance to complex and expensive nonlinear algorithms such as ANN and GBM. This approach also resolved the “black box” issue of ANN and GBM, improving clinical utility by identifying predominant features contributing to ACS. Furthermore, methods such as discriminant analysis, Naive Bayes, logistic and Cox regression, and classification provide relationships between predictors and outcomes, enabling risk scores to be calculated for each predictor in clinical practice [160]. In addition, there has recently been an increasing focus on explaining AI algorithms, with Xuan et al [97] developing a visual analytics tool for comparing convolutional neural networks (CNNs) to support in-depth inspection and comparative studies of CNN models. Wang et al [98] have reported a CNN explainer, which can show the CNN’s structure and provides on-demand dynamic visual explanations. Furthermore, it is exciting that explainable AI techniques have been applied to CVD prediction models and are reasonably expected to promote their application [161,162].

### AI-Ethics Pitfalls Also Should Be Considered During the Study Design

Informed model selection is a pivotal decision-making step in AI, particularly when dealing with CVD risk prediction. It is imperative to initiate the process with a rigorous assessment of bias and fairness [144]. While it is acknowledged that most AI algorithms may inherently carry biases, it is equally important to benchmark these biases against those prevalent in the existing systems. AI’s propensity to introduce bias often arises from making unequal errors across diverse demographic groups. The degree to which key demographic variables, including sex, age, and ethnicity, are adequately represented in the data set and incorporated during algorithm development significantly impacts the predictive accuracy across subgroups. Consequently, when these AI-derived predictions inform individual decisions, they can either perpetuate or intensify the existing disparities [163]. This issue is exacerbated by data that may not authentically mirror the entire target population, underscoring the ethical imperative of ensuring fairness at every stage of the project life cycle [164]. In the domain of CVD risk prediction, these ethical considerations loom large. For example, Kim et al [99] and Weng et al [71] constructed AI models for CVD risk prediction in the Korea National Health and Nutrition Examination Survey-VI cohort (4244 participants) and the CPRD cohort (378,256 participants), respectively. However, they failed to perform sensitivity analysis for race and gender subgroups, leading to gender and ethnic bias. These issues will significantly impact the application of the models.

There is an undeniable global demand for the development of AI systems that can be relied upon for their trustworthiness [165-168]. This demand is underscored by real-world examples, such as ProPublica’s investigation into the Correctional Offender Management Profiling for Alternative Sanctions software, which serves as a stark illustration of how algorithmic performance can exhibit disparities rooted in racial factors. Notably, the field of fairness evaluation has made significant methodological advancements that directly facilitate such analyses [169]. It is imperative for AI developers and health care professionals to actively engage with these tools. Researchers can effectively showcase bias in critical subgroups, such as minority ethnic communities or distinct age groups, through explicit presentation of these findings. This approach not only enhances transparency but also empowers users of the AI algorithm with the knowledge of its strengths and weaknesses in different demographic contexts. This consideration holds particular relevance in the domain of AI-CVD risk prediction. Segar et al [100] included 19,080 participants from Atherosclerosis Risk in Communities, Dallas Heart Study, Jackson Heart Study, and MESA cohort to build 6 AI models for predicting incident HF. They performed sensitivity analysis for the subgroup of race and sex, and all models performed well.

## Conclusions

In conclusion, we highlight that the effectiveness and application of ML models for predicting CVD risk are heavily reliant on data quality, data set characteristics, model design and statistical methods, and clinical implications. To address these challenges, we propose practical solutions, including gathering objective data, improving training, repeating measurements, increasing sample size, preventing overfitting using statistical methods, using specific AI algorithms to address targeted issues, standardizing outcomes and evaluation criteria, and enhancing fairness and replicability. It may provide a helpful reference and assistance to researchers, algorithm developers, policy makers, and clinical practitioners in the ML field of CVD prediction.

## Appendix

Multimedia Appendix 1 Search strategies of artificial intelligence or machine learning assessment guidelines or tools.

Multimedia Appendix 2 The evaluation content of assessment guidelines or tools in the field of medical artificial intelligence or machine learning research.

Multimedia Appendix 3 The framework for pitfalls in developing machine learning models for cardiovascular disease prediction.

## Abbreviations

ACM: all-cause mortality
ACS: acute coronary syndrome
AI-TREE: AI transparent, replicable, ethical, and effective research
AI: artificial intelligence
ANLL: average negative log likelihood
ANN: artificial neural network
ASCVD: atherosclerotic cardiovascular diseases
AUC: area under the curve
BP: blood pressure
CHD: coronary heart disease
CNN: convolutional neural network
CONSORT: Consolidated Standards of Reporting Trials
CPRD: Clinical Practice Research Datalink
CVD: cardiovascular disease
ECG: electrocardiogram
EHR: electronic health record
FRS: Framingham risk score
GBM: gradient boosting machine
HF: heart failure
ICD: International Classification of Diseases
KNN: k-nearest neighbor
LR: logistic regression
MESA: Multi-Ethnic Study of Atherosclerosis
MI: myocardial infarction
ML: machine learning
NHIS-HEALS: National Health Insurance System-National Health Screening Cohort
PCE: pooled cohort equation
PROBAST: Prediction Model Risk of Bias Assessment Tool
RF: random forest
RSF: random survival forest
SBP: systolic blood pressure
SPRINT: Systolic Blood Pressure Intervention Trial
SVM: support vector machine
TRIPOD: Transparent Reporting of a Multivariable Prediction Model for Individual Prognosis Or Diagnosis
