# Supplementary material for: Pitfalls in Developing Machine Learning Models for Predicting Cardiovascular Diseases: Challenge and Solutions
Source: J Med Internet Res. 2024 Jul 26;26:e47645. doi: 10.2196/47645 (PMC11316160; doi:10.2196/47645)
Supplement: Multimedia Appendix 1 [file jmir_v26i1e47645_app1.docx]

Supplementary material 1: Search strategies of AI/ML assessment guidelines or tools.

**Search in Pubmed**

Assessment tool [Text Word] OR Assessment score [Text Word] OR Guideline [Text Word] OR Criteria [Text Word] OR Checklist [Text Word] OR Framework [Text Word]

AND

Machine learning [MeSH Terms] OR Artificial intelligence [MeSH Terms] OR Machine learning [Text Word] OR Artificial intelligence [Text Word]

AND

Prediction [Text Word] OR Validation [Text Word] OR Risk [MeSH Terms] OR Bias [MeSH Terms] OR Risk [Text Word] OR Bias [Text Word]

AND

"1985/01/01"[Date - Publication]: "2024/2/29"[Date - Publication]
