# Supplementary material for: Pitfalls in Developing Machine Learning Models for Predicting Cardiovascular Diseases: Challenge and Solutions
Source: J Med Internet Res. 2024 Jul 26;26:e47645. doi: 10.2196/47645 (PMC11316160; doi:10.2196/47645)
Supplement: Multimedia Appendix 2 [file jmir_v26i1e47645_app2.docx]

Supplementary material 2: The evaluation content of assessment guidelines or tools in the field of medical AI/ML research.

| **No.^a^** | **Abbreviation** | **Year** | **Target Type** | **Evaluation Content (Grade Ⅰ)** | **Evaluation Content (Grade II)** | **Evaluation Quality Type** |
| --- | --- | --- | --- | --- | --- | --- |
| [1]  Tanguay et al. | - | 2023 | Radiology AI software | Company information | company information | Developing |
|  |  |  |  |  | contact information | Developing |
|  |  |  |  | Clinical information about the product | product description | Developing |
|  |  |  |  |  | target population | Developing |
|  |  |  |  |  | modality | Developing |
|  |  |  |  |  | organ | Developing |
|  |  |  |  |  | classes of use cases | Developing |
|  |  |  |  |  | role in clinical workflow | Developing |
|  |  |  |  |  | user interaction | Developing |
|  |  |  |  |  | type of application | Developing |
|  |  |  |  |  | product performance metrics | Developing |
|  |  |  |  | Engineering information about the product |  | Developing |
|  |  |  |  | Information on product development and status | source | Developing |
|  |  |  |  |  | total sample size | Developing |
|  |  |  |  |  | number of sites | Developing |
|  |  |  |  |  | countries of origin | Developing |
|  |  |  |  |  | clinical validation | Developing |
|  |  |  |  |  | regulatory approval | Developing |
|  |  |  |  | Demonstration |  | Developing |
|  |  |  |  | Information on cost |  | Developing |
|  |  |  |  | Relevant scientific publications |  | Developing |
| [2]  Lennerz et al. | DQM | 2023 | Diagnostic models | Care team and patient perspective | baseline | Developing |
|  |  |  |  |  | responsibility | Developing |
|  |  |  |  |  | assurance of patient safety | Developing |
|  |  |  |  |  | applicability and bias | Developing |
|  |  |  |  |  | beneficence | Developing |
|  |  |  |  | Data and measurements | methods of measurement | Developing |
|  |  |  |  |  | data sources and harmonization of data | Developing |
|  |  |  |  |  | data characterization | Developing |
|  |  |  |  |  | data standardization | Developing |
|  |  |  |  |  | uncertainty measure | Developing |
|  |  |  |  |  | electronic monitoring | Developing |
|  |  |  |  |  | prioritization of results | Developing |
|  |  |  |  | Ground truth assessment | identification of errors | Developing |
|  |  |  |  |  | diagnosis | Developing |
|  |  |  |  |  | demographic disparities | Developing |
|  |  |  |  | Health information technology | information-gathering | Developing |
|  |  |  |  |  | information synthesis | Developing |
|  |  |  |  |  | detecting safety risks | Developing |
|  |  |  |  |  | laboratory information system (LIS) | Developing |
|  |  |  |  |  | EHR integration | Developing |
|  |  |  |  | Organizational factors | teamwork | Developing |
|  |  |  |  |  | leadership | Developing |
|  |  |  |  |  | development methodology | Developing |
|  |  |  |  |  | strategies, interventions, timeline | Developing |
|  |  |  |  |  | personnel | Developing |
|  |  |  |  |  | competency | Developing |
|  |  |  |  |  | effort and capabilities | Developing |
|  |  |  |  |  | synergy | Developing |
|  |  |  |  |  | integration with existing quality management system | Developing |
|  |  |  |  | Regulatory aspects | analytical validity (AV) | Developing |
|  |  |  |  |  | clinical validity (CV) | Developing |
|  |  |  |  |  | patient safety | Developing |
|  |  |  |  |  | intended use | Developing |
|  |  |  |  |  | indication for use | Developing |
|  |  |  |  |  | proficiency testing | Developing |
|  |  |  |  |  | AI/ML performance monitoring | Developing |
|  |  |  |  |  | performance drift | Developing |
|  |  |  |  | Reimbursement | implementation cost | Developing |
|  |  |  |  |  | ongoing cost | Developing |
|  |  |  |  |  | clinical utility (CU) | Developing |
|  |  |  |  |  | applicable diagnoses | Developing |
|  |  |  |  |  | billing code | Developing |
|  |  |  |  |  | payor policies | Developing |
| [3]  Cerda-Alberich et al. | MAIC-10 | 2023 | Medical images models | Introduction | clinical need | Reporting |
|  |  |  |  | Materials and methods | study design | Reporting |
|  |  |  |  |  | safety and privacy | Reporting |
|  |  |  |  |  | data curation | Reporting |
|  |  |  |  |  | data annotation (ground truth) | Reporting |
|  |  |  |  |  | data partitioning (splitting) | Reporting |
|  |  |  |  |  | AI model | Reporting |
|  |  |  |  | Results, discussion | robustness | Reporting |
|  |  |  |  | Discussion | explainability | Reporting |
|  |  |  |  |  | transparency | Reporting |
| [4]  Sujan et al. | BS30440 | 2023 | The use of AI in healthcare | Inception | healthcare need | Developing |
|  |  |  |  | Development | model development | Developing |
|  |  |  |  |  | stakeholder involvement | Developing |
|  |  |  |  |  | training data | Developing |
|  |  |  |  |  | human factors /ergonomics | Developing |
|  |  |  |  |  | carbon impact | Developing |
|  |  |  |  | Validation | clinical effectiveness | Developing |
|  |  |  |  |  | external validity | Developing |
|  |  |  |  |  | equity and bias | Developing |
|  |  |  |  | Deployment | resourcing | Developing |
|  |  |  |  |  | cost and resource impact | Developing |
|  |  |  |  |  | assessing security vulnerabilities | Developing |
|  |  |  |  |  | patient safety | Developing |
|  |  |  |  |  | explainability | Developing |
|  |  |  |  |  | fair commercialisation | Developing |
|  |  |  |  | Monitoring | routine monitoring | Developing |
|  |  |  |  |  | product process modification | Developing |
|  |  |  |  |  | decommission | Developing |
| [5]  Mylrea and Robinson | AI-TMM | 2023 | Trustworth | Explainability / explainable artificial intelligence | feature importance analysis | Developing |
|  |  |  |  |  | lime (local interpretable model-agnostic explanations) | Developing |
|  |  |  |  |  | counterfactual analysis | Developing |
|  |  |  |  |  | model distillation | Developing |
|  |  |  |  |  | shapley additive explanations | Developing |
|  |  |  |  |  | attention visualization | Developing |
|  |  |  |  | Data privacy | data privacy metrics | Developing |
|  |  |  |  | Technical robustness and safety | adversarial robustness | Developing |
|  |  |  |  |  | generalization performance | Developing |
|  |  |  |  |  | stability and sensitivity analysis | Developing |
|  |  |  |  |  | error analysis | Developing |
|  |  |  |  |  | coverage and edge cases | Developing |
|  |  |  |  |  | safety constraints and compliance | Developing |
|  |  |  |  |  | failure modes and risk analysis | Developing |
|  |  |  |  | Transparency | explainability methods | Developing |
|  |  |  |  |  | model complexity | Developing |
|  |  |  |  |  | intelligibility of output | Developing |
|  |  |  |  |  | documentation and annotations | Developing |
|  |  |  |  |  | user feedback | Developing |
|  |  |  |  | Data use and design | data bias analysis | Developing |
|  |  |  |  |  | data privacy compliance | Developing |
|  |  |  |  |  | data governance | Developing |
|  |  |  |  |  | ethical data sourcing | Developing |
|  |  |  |  |  | human-in-the-loop evaluation | Developing |
|  |  |  |  | Societal well being | bias mitigation | Developing |
|  |  |  |  |  | inclusivity and diversity | Developing |
|  |  |  |  |  | ethical guidelines | Developing |
|  |  |  |  |  | user safety measures | Developing |
|  |  |  |  |  | accountability | Developing |
|  |  |  |  |  | transparency | Developing |
|  |  |  |  | Accountability | model performance monitoring | Developing |
|  |  |  |  |  | bias detection and mitigation | Developing |
|  |  |  |  |  | explainability and interpretability | Developing |
|  |  |  |  |  | transparency and auditing | Developing |
|  |  |  |  |  | error analysis and feedback mechanisms | Developing |
| [6]  Kwong et al. | APPRAISE-AI | 2023 | Clinical decision support | Clinical relevance | title | Reporting |
|  |  |  |  |  | background | Reporting |
|  |  |  |  |  | objective and problem | Reporting |
|  |  |  |  |  | implementation into clinical practice | Reporting |
|  |  |  |  | Data quality | source of data | Developing |
|  |  |  |  |  | eligibility criteria | Developing |
|  |  |  |  |  | ground truth | Developing |
|  |  |  |  |  | data abstraction, cleaning, preparation | Developing |
|  |  |  |  | Methodological conduct | sample size calculation | Developing |
|  |  |  |  |  | data splitting | Developing |
|  |  |  |  |  | baseline | Developing |
|  |  |  |  | Robustness of results | model evaluation | Developing |
|  |  |  |  |  | bias assessment | Reporting |
|  |  |  |  |  | model explanation | Reporting |
|  |  |  |  |  | clinical utility assessment | Reporting |
|  |  |  |  |  | error analysis | Reporting |
|  |  |  |  | Reporting quality | critical analysis | Reporting |
|  |  |  |  |  | cohort characteristics | Reporting |
|  |  |  |  |  | limitations | Reporting |
|  |  |  |  |  | disclosures | Reporting |
|  |  |  |  | Replicability | model and processing description | Reporting |
|  |  |  |  |  | hyperparameter tuning | Reporting |
|  |  |  |  |  | model specification | Reporting |
|  |  |  |  |  | transparency | Reporting |
| [7]  Klement and El Emam | Consolidated Guidelines | 2023 | Prognostic and diagnostic models | Study details | the medical/clinical task of interest | Reporting |
|  |  |  |  |  | the research question | Reporting |
|  |  |  |  |  | current medical/clinical practice | Reporting |
|  |  |  |  |  | the known predictors and confounders to what is being predicted / diagnosed | Reporting |
|  |  |  |  |  | the overall study design | Reporting |
|  |  |  |  |  | the medical institutional setting(s) | Reporting |
|  |  |  |  |  | the target patient population | Reporting |
|  |  |  |  |  | the intended use of the ML model | Reporting |
|  |  |  |  |  | existing model performance benchmarks for this task | Reporting |
|  |  |  |  |  | ethical and other regulatory approvals obtained | Reporting |
|  |  |  |  | Data | inclusion / exclusion criteria for the patient cohort | Reporting |
|  |  |  |  |  | methods of data collection | Reporting |
|  |  |  |  |  | bias introduced due to the method of data collection used | Reporting |
|  |  |  |  |  | methods of data transformations and preprocessing applied | Reporting |
|  |  |  |  |  | sample size calculation | Reporting |
|  |  |  |  |  | known quality issues with the data | Reporting |
|  |  |  |  |  | data availability | Reporting |
|  |  |  |  |  | data characteristics | Reporting |
|  |  |  |  | Methodology | strategies for handling missing data | Reporting |
|  |  |  |  |  | strategies for addressing class imbalance | Reporting |
|  |  |  |  |  | strategies for reducing dimensionality of data | Reporting |
|  |  |  |  |  | strategies for handling outliers | Reporting |
|  |  |  |  |  | strategies for data augmentation | Reporting |
|  |  |  |  |  | strategies for model pre-training | Reporting |
|  |  |  |  |  | the rationale for selecting the machine learning algorithm | Reporting |
|  |  |  |  |  | the method of evaluating model performance during training | Reporting |
|  |  |  |  |  | the method used for hyperparameter tuning | Reporting |
|  |  |  |  |  | model’s output adjustments | Reporting |
|  |  |  |  | Model evaluation | performance metrics used to evaluate the model | Reporting |
|  |  |  |  |  | the cost or consequence of errors | Reporting |
|  |  |  |  |  | the results of internal validation | Reporting |
|  |  |  |  |  | the final model hyperparameters | Reporting |
|  |  |  |  |  | model evaluation on an external dataset | Reporting |
|  |  |  |  |  | characteristics relevant for detecting data shift and drift | Reporting |
|  |  |  |  | Model explainability | plausibility of model outputs | Reporting |
|  |  |  |  |  | interpretation of model's results by an end-user | Reporting |
| [8]  Kocak et al. | CLEAR | 2023 | Radiomics research | Title | relevant title, specifying the radiomic methodology | Reporting |
|  |  |  |  | Abstract | structured summary with relevant information | Reporting |
|  |  |  |  | Keywords | relevant keywords for radiomics | Reporting |
|  |  |  |  | Introduction | scientific or clinical background | Reporting |
|  |  |  |  |  | rationale for using a radiomic approach | Reporting |
|  |  |  |  |  | study objective(s) | Reporting |
|  |  |  |  | Study design(method) | adherence to guidelines or checklists | Reporting |
|  |  |  |  |  | ethical details | Reporting |
|  |  |  |  |  | sample size calculation | Reporting |
|  |  |  |  |  | study nature (e.g., retrospective, prospective) | Reporting |
|  |  |  |  |  | eligibility criteria | Reporting |
|  |  |  |  |  | flowchart for technical pipeline | Reporting |
|  |  |  |  | Data(method) | data source (e.g., private, public) | Reporting |
|  |  |  |  |  | data overlap | Reporting |
|  |  |  |  |  | data split methodology | Reporting |
|  |  |  |  |  | imaging protocol (i.e., image acquisition and processing) | Reporting |
|  |  |  |  |  | definition of non-radiomic predictor variables | Reporting |
|  |  |  |  |  | definition of the reference standard | Reporting |
|  |  |  |  | Segmentation(method) | segmentation strategy | Reporting |
|  |  |  |  |  | details of operators performing segmentation | Reporting |
|  |  |  |  | Pre-processing(method) | image pre-processing details | Reporting |
|  |  |  |  |  | resampling method and its parameters | Reporting |
|  |  |  |  |  | discretization method and its parameters | Reporting |
|  |  |  |  |  | image types (e.g., original, filtered, transformed) | Reporting |
|  |  |  |  | Feature extraction(method) | feature extraction method | Reporting |
|  |  |  |  |  | feature classes | Reporting |
|  |  |  |  |  | number of features | Reporting |
|  |  |  |  |  | default configuration statement for remaining parameters | Reporting |
|  |  |  |  | Data preparation(method) | handling of missing data | Reporting |
|  |  |  |  |  | details of class imbalance | Reporting |
|  |  |  |  |  | details of segmentation reliability analysis | Reporting |
|  |  |  |  |  | feature scaling details (e.g., normalization, standardization) | Reporting |
|  |  |  |  |  | dimension reduction details | Reporting |
|  |  |  |  | Modeling(method) | algorithm details | Reporting |
|  |  |  |  |  | training and tuning details | Reporting |
|  |  |  |  |  | handling of confounders | Reporting |
|  |  |  |  |  | model selection strategy | Reporting |
|  |  |  |  | Evaluation(method) | testing technique (e.g., internal, external) | Reporting |
|  |  |  |  |  | performance metrics and rationale for choosing | Reporting |
|  |  |  |  |  | uncertainty evaluation and measures (e.g., confidence intervals) | Reporting |
|  |  |  |  |  | statistical performance comparison (e.g., DeLong's test) | Reporting |
|  |  |  |  |  | comparison with non-radiomic and combined methods | Reporting |
|  |  |  |  |  | interpretability and explainability methods | Reporting |
|  |  |  |  | Results | baseline demographic and clinical characteristics | Reporting |
|  |  |  |  |  | flowchart for eligibility criteria | Reporting |
|  |  |  |  |  | feature statistics (e.g., reproducibility, feature selection) | Reporting |
|  |  |  |  |  | model performance evaluation | Reporting |
|  |  |  |  |  | comparison with non-radiomic and combined approaches | Reporting |
|  |  |  |  | Discussion | overview of important findings | Reporting |
|  |  |  |  |  | previous works with differences from the current study | Reporting |
|  |  |  |  |  | practical implications | Reporting |
|  |  |  |  |  | strengths and limitations (e.g., bias and generalizability issues) | Reporting |
|  |  |  |  | Open science | data availability | Reporting |
|  |  |  |  |  | code availability | Reporting |
|  |  |  |  |  | model availability | Reporting |
| [9]  van Smeden et al. | - | 2022 | Prediction models | Conceptualization | AI prediction model needed | Developing |
|  |  |  |  |  | fit in the intended clinical warkflow | Developing |
|  |  |  |  | Data collection | sample size | Developing |
|  |  |  |  |  | representative | Developing |
|  |  |  |  | Predictors and outcome | measure outcome reliably | Developing |
|  |  |  |  |  | measure predictors at baseline | Developing |
|  |  |  |  | Openness and fairness | algorithmic fairness | Developing |
|  |  |  |  |  | open for use and testing | Developing |
|  |  |  |  | Reporting | follow reporting guidelines | Reporting |
|  |  |  |  |  | prediction time horizon | Reporting |
|  |  |  |  | Model performance | internal/external validation | Developing |
|  |  |  |  |  | performance evaluation | Developing |
| [10]  Daneshjou et al. | CLEAR Derm | 2022 | Medical images models | Data | metadata on images and potential biases | Reporting |
|  |  |  |  |  | imaging modalities | Reporting |
|  |  |  |  |  | confounding artifacts | Reporting |
|  |  |  |  |  | pre/post data processing | Reporting |
|  |  |  |  |  | define image datasets (training, validation, test) | Reporting |
|  |  |  |  |  | relation of test dataset and proposed clinical setting | Reporting |
|  |  |  |  | Technique | algorithms using standard labels of reference | Reporting |
|  |  |  |  |  | algorithm development | Reporting |
|  |  |  |  |  | public evaluation method for AI algorithm or algorithm output | Reporting |
|  |  |  |  | Technical assessment | publicly evaluate algorithm | Reporting |
|  |  |  |  |  | performance measures | Reporting |
|  |  |  |  |  | benchmarking, technical comparison, and novelty | Reporting |
|  |  |  |  |  | bias assessment | Reporting |
|  |  |  |  | Application | use cases and target conditions (inside distribution) | Reporting |
|  |  |  |  |  | potential impacts on the healthcare team and patients | Reporting |
| [11]  Vasey et al. | DECIDE-AI | 2022 | Decision support systems | Title and abstract |  | Reporting |
|  |  |  |  | Intended use(introduction) | targeted medical condition(s) and problem(s) | Reporting |
|  |  |  |  |  | intended users of the AI system, its planned integration in the care pathway, and the potential effect | Reporting |
|  |  |  |  | Participants(method) | patients recruition | Reporting |
|  |  |  |  |  | users recruition | Reporting |
|  |  |  |  |  | familiarize the users with the AI system | Reporting |
|  |  |  |  | Al system(method) | describe the AI system | Reporting |
|  |  |  |  |  | input data | Reporting |
|  |  |  |  |  | AI system outputs | Reporting |
|  |  |  |  | Implementation(method) | implementation | Reporting |
|  |  |  |  | Outcomes(method) | outcomes | Reporting |
|  |  |  |  | Safety and errors(method) | safety and errors | Reporting |
|  |  |  |  | Human factors(method) | human factors | Reporting |
|  |  |  |  | Analysis(method) | analysis | Reporting |
|  |  |  |  | Ethics(method) | ethics | Reporting |
|  |  |  |  | Patient involvement(method) | patient involvement | Reporting |
|  |  |  |  | Participants(result) | input data missingness | Reporting |
|  |  |  |  |  | baseline characteristics | Reporting |
|  |  |  |  | Implementation(result) | implementation | Reporting |
|  |  |  |  | Main results(result) | main results | Reporting |
|  |  |  |  | Subgroups analysis(result) | subgroups analysis | Reporting |
|  |  |  |  | Modifications(result) | modifications | Reporting |
|  |  |  |  | Human–computer agreement(result) | human–computer agreement | Reporting |
|  |  |  |  | Safety and errors(result) | safety and errors | Reporting |
|  |  |  |  | Human factors(result) | human factors | Reporting |
|  |  |  |  | Discussion | support for intended use | Reporting |
|  |  |  |  |  | safety and errors | Reporting |
|  |  |  |  |  | strengths and limitations | Reporting |
|  |  |  |  | Statements | data availability | Reporting |
|  |  |  |  |  | conflicts of interest | Reporting |
| [12]  Jha et al. | RELAINCE Guidelines | 2022 | AI models | Technical task-specific evaluation | technical task-specific evaluation | Developing |
|  |  |  |  | Proof of concept evaluation | proof of concept evaluation | Reporting |
|  |  |  |  | Postdeployment evaluation | postdeployment evaluation | Reporting |
|  |  |  |  | Clinical evaluation | clinical evaluation | Developing |
| [13]  Banerjee et al. | - | 2021 | Prediction models | Clinical relevance (development) | patient benefit | Developing |
|  |  |  |  |  | target condition applicability | Developing |
|  |  |  |  |  | data suitability | Developing |
|  |  |  |  | Patients (development) | patient applicability | Developing |
|  |  |  |  |  | selection bias | Developing |
|  |  |  |  | Algorithm (development) | algorithm applicability | Developing |
|  |  |  |  |  | bias in algorithm | Developing |
|  |  |  |  | Validation | internal | Developing |
|  |  |  |  |  | external | Developing |
|  |  |  |  | Clinical utility (impact) | improved prediction | Reporting |
|  |  |  |  |  | methods available | Reporting |
|  |  |  |  |  | metrics relevant | Reporting |
|  |  |  |  |  | interpretable | Reporting |
|  |  |  |  |  | results justified | Reporting |
|  |  |  |  | Effectiveness(impact) | real world | Reporting |
|  |  |  |  |  | cost effectiveness | Reporting |
| [14]  Walsh et al. | DOME | 2021 | Supervised ML models | Data | provenance | Reporting |
|  |  |  |  |  | data splits | Developing |
|  |  |  |  |  | redundancy between data splits | Developing |
|  |  |  |  |  | availability of data | Developing |
|  |  |  |  | Optimization | algorithm | Developing |
|  |  |  |  |  | meta-predictions | Developing |
|  |  |  |  |  | data encoding | Developing |
|  |  |  |  |  | availability of configuration | Developing |
|  |  |  |  |  | parameters | Developing |
|  |  |  |  |  | fitting | Developing |
|  |  |  |  |  | regularization | Developing |
|  |  |  |  | Model | output | Reporting |
|  |  |  |  |  | interpretability | Reporting |
|  |  |  |  |  | execution time | Reporting |
|  |  |  |  |  | availability of software | Reporting |
|  |  |  |  | Evaluation | evaluation method | Reporting |
|  |  |  |  |  | performance measures | Reporting |
|  |  |  |  |  | comparison | Reporting |
|  |  |  |  |  | confidence | Reporting |
|  |  |  |  |  | availability of evaluation | Reporting |
| [15]  Olczak et al. | CAIR | 2021 | Clinical AI research | Title and abstract | include that the method contains or uses an AI/ML | Reporting |
|  |  |  |  |  | AI tool’s intended use or purpose | Reporting |
|  |  |  |  | Introduction | clinical problem | Reporting |
|  |  |  |  | Method | inclusion and exclusion criteria | Reporting |
|  |  |  |  |  | how the input data was acquired, selected | Reporting |
|  |  |  |  |  | human–AI interaction | Reporting |
|  |  |  |  |  | specifications, design, and the parameters | Reporting |
|  |  |  |  |  | missing or poor-quality data | Reporting |
|  |  |  |  |  | outcome performance measures | Reporting |
|  |  |  |  |  | specific version of the AI model | Reporting |
|  |  |  |  |  | how the output contributed to decision-making and evaluation of the model | Reporting |
|  |  |  |  |  | output of the AI | Reporting |
|  |  |  |  | Results | result analysis | Reporting |
|  |  |  |  |  | performance errors | Reporting |
|  |  |  |  | Discussion and other information | ethical considerations and methodological biases | Reporting |
|  |  |  |  |  | AI model/data accessible | Reporting |
| [16]  Matschinske et al. | AIMe | 2021 | AI research | Metadata | metadata | Reporting |
|  |  |  |  | Purpose | what is your AI designed to learn or predict? | Reporting |
|  |  |  |  |  | surrogate marker | Reporting |
|  |  |  |  |  | category | Reporting |
|  |  |  |  | Data | what is the type of the data? | Reporting |
|  |  |  |  |  | origin | Reporting |
|  |  |  |  |  | availability | Reporting |
|  |  |  |  |  | data splitting | Reporting |
|  |  |  |  |  | biases | Reporting |
|  |  |  |  |  | samples and features | Reporting |
|  |  |  |  |  | pre-processing | Reporting |
|  |  |  |  | Method | which AI or mathematical methods did you use and how did you select them? | Reporting |
|  |  |  |  |  | how did you select your method’s hyper-parameters? | Reporting |
|  |  |  |  |  | test metrics | Reporting |
|  |  |  |  |  | overfitting | Reporting |
|  |  |  |  |  | trigger situations | Reporting |
|  |  |  |  |  | randomized steps | Reporting |
|  |  |  |  |  | baseline model | Reporting |
|  |  |  |  |  | state-of-the-art approaches | Reporting |
|  |  |  |  | Reproducibility | re-running | Reporting |
|  |  |  |  |  | source code | Reporting |
|  |  |  |  |  | pre-trained model | Reporting |
|  |  |  |  |  | execution environment | Reporting |
| [17]  Schwendicke et al. | - | 2021 | AI research | Planning and conducting | study goal | Developing |
|  |  |  |  |  | study focus | Developing |
|  |  |  |  |  | data | Developing |
|  |  |  |  |  | study aim | Developing |
|  |  |  |  |  | reference test | Developing |
|  |  |  |  |  | clustering | Developing |
|  |  |  |  |  | test dataset | Developing |
|  |  |  |  |  | computational resources | Developing |
|  |  |  |  |  | comparators | Developing |
|  |  |  |  | Reporting | title | Reporting |
|  |  |  |  |  | abstract | Reporting |
|  |  |  |  |  | introduction | Reporting |
|  |  |  |  |  | study design | Reporting |
|  |  |  |  |  | sampling(data) | Reporting |
|  |  |  |  |  | data protection(data) | Reporting |
|  |  |  |  |  | missing data(data) | Reporting |
|  |  |  |  |  | data processing(data) | Reporting |
|  |  |  |  |  | reference test | Reporting |
|  |  |  |  |  | sample size | Reporting |
|  |  |  |  |  | model parameters(model) | Reporting |
|  |  |  |  |  | training | Reporting |
|  |  |  |  |  | justify the best-performing model | Reporting |
|  |  |  |  |  | evaluation | Reporting |
|  |  |  |  |  | uncertainty | Reporting |
|  |  |  |  |  | explainability | Reporting |
|  |  |  |  |  | results | Reporting |
|  |  |  |  |  | performance metrics and data partitions(results) | Reporting |
|  |  |  |  |  | discussion | Reporting |
|  |  |  |  |  | other | Reporting |
| [18]  Scott et al. | - | 2021 | Clinical application | None | purpose and context | Developing |
|  |  |  |  |  | data | Developing |
|  |  |  |  |  | model performance | Developing |
|  |  |  |  |  | transferable | Developing |
|  |  |  |  |  | intelligible | Developing |
|  |  |  |  |  | clinical implication | Developing |
|  |  |  |  |  | ethics | Developing |
| [19]  Norgeot et al. | MI-CLAIM | 2020 | AI models | Study design | cohort characteristics | Reporting |
|  |  |  |  |  | clinical problem | Reporting |
|  |  |  |  |  | research question | Reporting |
|  |  |  |  | Data and optimization | data source | Reporting |
|  |  |  |  |  | data transformations | Reporting |
|  |  |  |  |  | data splitting | Reporting |
|  |  |  |  |  | select the best model | Reporting |
|  |  |  |  |  | input data type (structured or unstructured) | Reporting |
|  |  |  |  | Model performance | algorithm performance evaluation metrics | Reporting |
|  |  |  |  |  | appropriate statistical significance | Reporting |
|  |  |  |  | Model examination | examination technique | Reporting |
|  |  |  |  |  | relevance of the examination results with respect to model/algorithm performance | Reporting |
|  |  |  |  |  | interpretability | Reporting |
|  |  |  |  |  | data distribution | Reporting |
|  |  |  |  | Replicability | transparency | Reporting |
| [20]  Liu et al. | CONSORT-AI Extension | 2020 | Clinical trial reports | Title and abstract |  | Reporting |
|  |  |  |  | Introduction | background and objectives | Reporting |
|  |  |  |  | Methods | trial design | Reporting |
|  |  |  |  |  | participants | Reporting |
|  |  |  |  |  | interventions | Reporting |
|  |  |  |  |  | outcomes(methods) | Reporting |
|  |  |  |  |  | sample size(methods) | Reporting |
|  |  |  |  | Randomisation | sequence generation | Reporting |
|  |  |  |  |  | allocation concealment mechanism | Reporting |
|  |  |  |  |  | implementation | Reporting |
|  |  |  |  |  | blinding | Reporting |
|  |  |  |  |  | statistical methods | Reporting |
|  |  |  |  | Results | participant flow (a diagram is strongly recommended) | Reporting |
|  |  |  |  |  | recruitment | Reporting |
|  |  |  |  |  | baseline data | Reporting |
|  |  |  |  |  | numbers analysed | Reporting |
|  |  |  |  |  | outcomes and estimation | Reporting |
|  |  |  |  |  | ancillary analyses | Reporting |
|  |  |  |  |  | harms | Reporting |
|  |  |  |  | Discussion | limitations | Reporting |
|  |  |  |  |  | generalisability | Reporting |
|  |  |  |  |  | interpretation | Reporting |
|  |  |  |  | Other information | registration | Reporting |
|  |  |  |  |  | protocol | Reporting |
|  |  |  |  |  | funding | Reporting |
| [21]  Vollmer et al. | AI-TREE | 2020 | ML/AI health research | Inception | health question | Reporting |
|  |  |  |  |  | best practices in clinical research and epidemiological study design | Reporting |
|  |  |  |  | Study | data collection, analysis, deployment, and use | Developing |
|  |  |  |  |  | data quality | Developing |
|  |  |  |  |  | validation methodology | Developing |
|  |  |  |  |  | computational and software resources | Developing |
|  |  |  |  | Statistical methods | performance metrics | Reporting |
|  |  |  |  |  | compared to the current best technology | Reporting |
|  |  |  |  |  | gain in statistical performance | Reporting |
|  |  |  |  | Replicability | data accessible | Reporting |
|  |  |  |  |  | code, software, and all other relevant parts accessible | Reporting |
|  |  |  |  |  | organisational transparency | Reporting |
|  |  |  |  | Impact evaluation | generalisability | Reporting |
|  |  |  |  |  | AI-ethics | Reporting |
|  |  |  |  |  | interpretability | Reporting |
|  |  |  |  |  | how will evidence of real world model effectiveness in the proposed clinical setting be generated, and how will unintended consequences be prevented? | Reporting |
|  |  |  |  | Implementation | post-deployment monitoring | Reporting |
|  |  |  |  |  | cost effectiveness | Reporting |
|  |  |  |  |  | potential financial benefits distribution | Reporting |
|  |  |  |  |  | regulatory requirements for accreditation/approval | Reporting |
| [22]  Sengupta et al. | PRIME | 2020 | Medical images models | Designing study plan | need for ML models | Reporting |
|  |  |  |  |  | goals/objectives | Reporting |
|  |  |  |  |  | summary statistics | Reporting |
|  |  |  |  |  | ML workflow | Reporting |
|  |  |  |  | Data standardization, feature engineering, and learning | format/describe data | Reporting |
|  |  |  |  |  | normalize variables | Reporting |
|  |  |  |  |  | impute missing data | Reporting |
|  |  |  |  |  | remove outliers | Reporting |
|  |  |  |  |  | balance class | Reporting |
|  |  |  |  |  | describe feature selection | Reporting |
|  |  |  |  | Selection of machine learning models | deine analysis goal | Reporting |
|  |  |  |  |  | identify ML task | Reporting |
|  |  |  |  |  | define use of simple / complex models | Reporting |
|  |  |  |  |  | benchmark complex models | Reporting |
|  |  |  |  | Model assessment | optimize model parameters | Reporting |
|  |  |  |  |  | train model using CV | Reporting |
|  |  |  |  |  | define ensemble methods (if used) | Reporting |
|  |  |  |  |  | identify model interpretability | Reporting |
|  |  |  |  | Model evaluation | clearly define training test and validation sets | Reporting |
|  |  |  |  |  | provide summary of model parameter's | Reporting |
|  |  |  |  |  | report class balancing measures for classification tasks | Reporting |
|  |  |  |  | Model replicability | consider sharing code or scripts on public repositories | Reporting |
|  |  |  |  |  | provide a data dictionary | Reporting |
|  |  |  |  |  | document detailing software and libraries | Reporting |
|  |  |  |  | Reporting limitations | report all assumptions,biases and limitations | Reporting |
|  |  |  |  |  | provide performance metrics on hold-out or external validation set | Reporting |
| [23]  Cruz Rivera et al. | SPIRIT-AI Extension | 2020 | Clinical AI intervention trials | Administrative information | title | Reporting |
|  |  |  |  |  | trial registration | Reporting |
|  |  |  |  |  | protocol version | Reporting |
|  |  |  |  |  | funding | Reporting |
|  |  |  |  |  | roles and responsibilities | Reporting |
|  |  |  |  | Introduction | background and rationale | Reporting |
|  |  |  |  |  | objectives | Reporting |
|  |  |  |  |  | trial design | Reporting |
|  |  |  |  |  | study setting | Reporting |
|  |  |  |  |  | eligibility criteria | Reporting |
|  |  |  |  |  | interventions | Reporting |
|  |  |  |  |  | outcomes | Reporting |
|  |  |  |  |  | participant timeline | Reporting |
|  |  |  |  |  | sample size | Reporting |
|  |  |  |  |  | recruitment | Reporting |
|  |  |  |  | Assignment of interventions (for controlled trials)(methods) | sequence generation | Reporting |
|  |  |  |  |  | allocation concealment mechanism | Reporting |
|  |  |  |  |  | implementation | Reporting |
|  |  |  |  |  | blinding (masking) | Reporting |
|  |  |  |  | Data collection, management, and analysis(methods) | data collection methods | Reporting |
|  |  |  |  |  | data management | Reporting |
|  |  |  |  |  | statistical methods | Reporting |
|  |  |  |  | Monitoring(methods) | data monitoring | Reporting |
|  |  |  |  |  | harms | Reporting |
|  |  |  |  |  | auditing | Reporting |
|  |  |  |  | Ethics and dissemination | protocol amendments | Reporting |
|  |  |  |  |  | consent or ascent | Reporting |
|  |  |  |  |  | confidentiality | Reporting |
|  |  |  |  |  | declaration of interests | Reporting |
|  |  |  |  |  | access to data | Reporting |
|  |  |  |  |  | ancillary and post-trial care | Reporting |
|  |  |  |  |  | dissemination policy | Reporting |
|  |  |  |  | Appendices | informed consent materials | Reporting |
|  |  |  |  |  | biological specimens | Reporting |
| [24]  Kakarmath et al. | - | 2020 | AI Research | Datasets | the datasets used for model development, validation, and testing should be adequately described | Reporting |
|  |  |  |  | Methods | provide a detailed description of the methods used for model development and testing | Reporting |
|  |  |  |  | Model performance | describe the model’s performance | Reporting |
|  |  |  |  | Limitations | discuss the limitations of the model and/or the methods used | Reporting |
|  |  |  |  | Implementation | the implications of errors made by the model on clinical and economic outcomes | Reporting |
|  |  |  |  |  | present clinical acceptability and user perceptions | Reporting |
|  |  |  |  |  | generalizability | Reporting |
| [25]  Stevens et al. | - | 2020 | Clinical AI research | Study design | intended use of results | Reporting |
|  |  |  |  |  | evaluation measures, training protocols, and validation | Reporting |
|  |  |  |  |  | clinical question | Reporting |
|  |  |  |  |  | problem type | Reporting |
|  |  |  |  |  | available data | Reporting |
|  |  |  |  |  | ML method and rationale | Reporting |
|  |  |  |  | Data source and preprocessing | population | Reporting |
|  |  |  |  |  | sample record and meaturement characteristics | Reporting |
|  |  |  |  |  | data splitting | Reporting |
|  |  |  |  |  | data collection and quality | Reporting |
|  |  |  |  |  | data processing | Reporting |
|  |  |  |  |  | link to data or data request mechanism | Reporting |
|  |  |  |  | Model development and validation | hardware, software, packages used | Reporting |
|  |  |  |  |  | model training and evaluation | Reporting |
|  |  |  |  |  | model parameters/hyperparameters | Reporting |
|  |  |  |  |  | feature selected and input | Reporting |
|  |  |  |  |  | validation method and performance metrics | Reporting |
|  |  |  |  |  | reproducibility and code reuse | Reporting |
| [26]  Wolff et al. | PROBAST | 2019 | Prediction model | Participants(risk of bias) | data source | Developing |
|  |  |  |  |  | inclusions and exclusions | Developing |
|  |  |  |  | Participants(application) | participants and clinical setting | Reporting |
|  |  |  |  | Predictors(risk of bias) | definition and assessment | Developing |
|  |  |  |  |  | blinding | Developing |
|  |  |  |  |  | data availability | Developing |
|  |  |  |  | Predictors(application) | definition, assessment, and timing of predictors | Reporting |
|  |  |  |  | Outcome(risk of bias) | method of outcome determination | Developing |
|  |  |  |  |  | objective, standard and prespecified | Developing |
|  |  |  |  |  | exclusion of predictors | Developing |
|  |  |  |  |  | definedor determined in a similar way | Developing |
|  |  |  |  |  | blinding | Developing |
|  |  |  |  |  | time interval | Developing |
|  |  |  |  | Outcome(application) | outcome definition, timing, or determination | Reporting |
|  |  |  |  | Analysis(risk of bias) | number of participants with the outcome | Developing |
|  |  |  |  |  | continuous and categorical predictors | Developing |
|  |  |  |  |  | incomplete participants | Developing |
|  |  |  |  |  | handle missing data | Developing |
|  |  |  |  |  | avoid univariable selection | Developing |
|  |  |  |  |  | account for complexities in the data | Developing |
|  |  |  |  |  | evaluation of calibration and discrimination | Developing |
|  |  |  |  |  | accounted for overfitting and optimism | Developing |
|  |  |  |  |  | same predictors and coefficients as the final presented multivariable analyses | Developing |
| [27]  Lambin et al. | RQS | 2017 | Radiomics research | Data selection | image protocol quality | Developing |
|  |  |  |  |  | multiple segmentations | Developing |
|  |  |  |  |  | phantom study on all scanners | Developing |
|  |  |  |  |  | imaging at multiple time points | Developing |
|  |  |  |  | Data processing methods | feature reduction or adjustment for multiple testing | Developing |
|  |  |  |  |  | multivariable analysis with non radiomics features (for example, EGFR mutation) | Developing |
|  |  |  |  |  | detect and discuss biological correlates | Developing |
|  |  |  |  |  | cut-off analyses | Developing |
|  |  |  |  | Statistical methods | discrimination statistics | Developing |
|  |  |  |  |  | calibration statistics | Developing |
|  |  |  |  |  | prospective study registered in a trial database | Developing |
|  |  |  |  |  | validation | Developing |
|  |  |  |  |  | comparison to ‘gold standard’ | Developing |
|  |  |  |  |  | potential clinical utility | Reporting |
|  |  |  |  | Information on cost | cost-effectiveness analysis | Reporting |
|  |  |  |  | Replicability | open science and data | Reporting |
| [28]  Luo et al. | - | 2016 | Prediction models | Title and abstract | nature of study(title) | Reporting |
|  |  |  |  |  | background(abstract) | Reporting |
|  |  |  |  |  | summary(abstract) | Reporting |
|  |  |  |  | Discussion section | clinical implications | Reporting |
|  |  |  |  |  | limitations of the model | Reporting |
|  |  |  |  |  | unexpected results during the experiments | Reporting |
|  |  |  |  | Introduction section | rationale | Reporting |
|  |  |  |  |  | objectives | Reporting |
|  |  |  |  | Methods section | define the prediction problem | Developing |
|  |  |  |  |  | describe the setting | Developing |
|  |  |  |  |  | prepare data for model building | Developing |
|  |  |  |  |  | build the predictive model | Developing |
|  |  |  |  | Results section | report the final model and performance | Reporting |
| [29]  Moons et al. | TRIPOD | 2015 | Prognosis or diagnosis models | Title(title and abstract) | multivariable prediction model, the target population, and the outcome to be predicted | Reporting |
|  |  |  |  | Abstract(title and abstract) | objectives, study design, setting, participants, sample size, predictors, outcome, statistical analysis, results, and conclusions | Reporting |
|  |  |  |  | Background and objectives(introduction) | medical context and rationale | Reporting |
|  |  |  |  |  | objectives | Reporting |
|  |  |  |  | Source of data(methods) | study design or source of data | Reporting |
|  |  |  |  |  | key study dates(timing) | Reporting |
|  |  |  |  | Participants(methods) | study setting | Reporting |
|  |  |  |  |  | eligibility criteria | Reporting |
|  |  |  |  |  | treatments received | Reporting |
|  |  |  |  | Outcome(methods) | definition | Reporting |
|  |  |  |  |  | blinding | Reporting |
|  |  |  |  | Predictors(methods) | definition | Reporting |
|  |  |  |  |  | blinding | Reporting |
|  |  |  |  | Sample size(methods) | sample size | Reporting |
|  |  |  |  | Missing data(methods) | missing data | Reporting |
|  |  |  |  | Statistical analysis methods(methods) | performance measures | Reporting |
|  |  |  |  |  | validation | Reporting |
|  |  |  |  |  | model updating | Reporting |
|  |  |  |  | Risk groups(methods) | risk groups | Reporting |
|  |  |  |  | Development vs. Validation(methods) | differencesin setting,eligibility criteria, outcome, and predictors | Reporting |
|  |  |  |  | Participants(results) | flow of participants through the study | Reporting |
|  |  |  |  |  | characteristics of participants | Reporting |
|  |  |  |  |  | distribution of important variables | Reporting |
|  |  |  |  | Model development(results) | unadjusted association between each candidate predictor and outcome | Reporting |
|  |  |  |  |  | number of participants and outcome events | Reporting |
|  |  |  |  | Model specification(results) | present the full prediction model | Reporting |
|  |  |  |  |  | explain how to use | Reporting |
|  |  |  |  | Model performance(results) | model performance | Reporting |
|  |  |  |  | Model updating(results) | model updating | Reporting |
|  |  |  |  | Limitations(discussion) | limitations | Reporting |
|  |  |  |  | Interpretation(discussion) | interpretation | Reporting |
|  |  |  |  | Implications(discussion) | potential clinical use and implications for future research | Reporting |
|  |  |  |  | Supplementary information(other information) | supplementary information | Reporting |
|  |  |  |  | Funding(other information) | funding | Reporting |
| [30]  Wilkinson et al. | FAIR | 2016 | Scientific data management and stewardship | Reusability | clear and accessible data usage license | Reporting |
|  |  |  |  |  | domain-relevant community standards | Reporting |
|  |  |  |  |  | detailed provenance | Reporting |
|  |  |  |  | Interoperability | knowledge representation language | Reporting |
|  |  |  |  |  | vocabularies that follow fair principles | Reporting |
|  |  |  |  |  | qualified references to other (meta) data | Reporting |
|  |  |  |  | Findability | assigned a globally unique and persistent identifier | Reporting |
|  |  |  |  |  | described with rich metadata | Reporting |
|  |  |  |  |  | include the identifier | Reporting |
|  |  |  |  |  | registered or indexed in a searchable resource | Reporting |
|  |  |  |  | Accessibility | standardized communications protocol | Reporting |
| [31]  van Royen et al. | Five critical quality criteria | 2023 | AI-based prediction models | Adequate sample size | adequate sample size | Developing |
|  |  |  |  | Rigorous validation | rigorous validation | Developing |
|  |  |  |  | Reporting and reproducibility | reporting and reproducibility | Reporting |
|  |  |  |  | Clear intended use | clear intended use | Reporting |
|  |  |  |  | Openness of data and software | openness of data and software | Reporting |

^a^No.: Number of guideline.

**References**

1. Tanguay, W., et al., *Assessment of Radiology Artificial Intelligence Software: A Validation and Evaluation Framework.* Can Assoc Radiol J, 2023. **74**(2): p. 326-333.

2. Lennerz, J.K., et al., *Diagnostic quality model (DQM): an integrated framework for the assessment of diagnostic quality when using AI/ML.* Clin Chem Lab Med, 2023. **61**(4): p. 544-557.

3. Cerda-Alberich, L., et al., *MAIC-10 brief quality checklist for publications using artificial intelligence and medical images.* Insights Imaging, 2023. **14**(1): p. 11.

4. Sujan, M., et al., *Validation framework for the use of AI in healthcare: overview of the new British standard BS30440.* BMJ Health & Care Informatics Online, 2023. **30**(1).

5. Mylrea, M. and N. Robinson, *Artificial Intelligence (AI) Trust Framework and Maturity Model: Applying an Entropy Lens to Improve Security, Privacy, and Ethical AI.* Entropy (Basel), 2023. **25**(10).

6. Kwong, J.C.C., et al., *APPRAISE-AI Tool for Quantitative Evaluation of AI Studies for Clinical Decision Support.* JAMA Netw Open, 2023. **6**(9): p. e2335377.

7. Klement, W. and K. El Emam, *Consolidated Reporting Guidelines for Prognostic and Diagnostic Machine Learning Modeling Studies: Development and Validation.* J Med Internet Res, 2023. **25**: p. e48763.

8. Kocak, B., et al., *CheckList for EvaluAtion of Radiomics research (CLEAR): a step-by-step reporting guideline for authors and reviewers endorsed by ESR and EuSoMII.* Insights Imaging, 2023. **14**(1): p. 75.

9. van Smeden, M., et al., *Critical appraisal of artificial intelligence-based prediction models for cardiovascular disease.* Eur Heart J, 2022. **43**(31): p. 2921-2930.

10. Daneshjou, R., et al., *Checklist for Evaluation of Image-Based Artificial Intelligence Reports in Dermatology: CLEAR Derm Consensus Guidelines From the International Skin Imaging Collaboration Artificial Intelligence Working Group.* JAMA Dermatol, 2022. **158**(1): p. 90-96.

11. Vasey, B., et al., *Reporting guideline for the early-stage clinical evaluation of decision support systems driven by artificial intelligence: DECIDE-AI.* Nat Med, 2022. **28**(5): p. 924-933.

12. Jha, A.K., et al., *Nuclear Medicine and Artificial Intelligence: Best Practices for Evaluation (the RELAINCE Guidelines).* J Nucl Med, 2022. **63**(9): p. 1288-1299.

13. Banerjee, A., et al., *Machine learning for subtype definition and risk prediction in heart failure, acute coronary syndromes and atrial fibrillation: systematic review of validity and clinical utility.* BMC Med, 2021. **19**(1): p. 85.

14. Walsh, I., et al., *DOME: recommendations for supervised machine learning validation in biology.* Nat Methods, 2021. **18**(10): p. 1122-1127.

15. Olczak, J., et al., *Presenting artificial intelligence, deep learning, and machine learning studies to clinicians and healthcare stakeholders: an introductory reference with a guideline and a Clinical AI Research (CAIR) checklist proposal.* Acta Orthop, 2021. **92**(5): p. 513-525.

16. Matschinske, J., et al., *The AIMe registry for artificial intelligence in biomedical research.* Nat Methods, 2021. **18**(10): p. 1128-1131.

17. Schwendicke, F., et al., *Artificial intelligence in dental research: Checklist for authors, reviewers, readers.* J Dent, 2021. **107**: p. 103610.

18. Scott, I., S. Carter, and E. Coiera, *Clinician checklist for assessing suitability of machine learning applications in healthcare.* BMJ Health Care Inform, 2021. **28**(1).

19. Norgeot, B., et al., *Minimum information about clinical artificial intelligence modeling: the MI-CLAIM checklist.* Nat Med, 2020. **26**(9): p. 1320-1324.

20. Liu, X., et al., *Reporting guidelines for clinical trial reports for interventions involving artificial intelligence: the CONSORT-AI extension.* Lancet Digit Health, 2020. **2**(10): p. e537-e548.

21. Vollmer, S., et al., *Machine learning and artificial intelligence research for patient benefit: 20 critical questions on transparency, replicability, ethics, and effectiveness.* BMJ, 2020. **368**: p. l6927.

22. Sengupta, P.P., et al., *Proposed Requirements for Cardiovascular Imaging-Related Machine Learning Evaluation (PRIME): A Checklist: Reviewed by the American College of Cardiology Healthcare Innovation Council.* JACC Cardiovasc Imaging, 2020. **13**(9): p. 2017-2035.

23. Cruz Rivera, S., et al., *Guidelines for clinical trial protocols for interventions involving artificial intelligence: the SPIRIT-AI extension.* Nat Med, 2020. **26**(9): p. 1351-1363.

24. Kakarmath, S., et al., *Best practices for authors of healthcare-related artificial intelligence manuscripts.* NPJ Digit Med, 2020. **3**: p. 134.

25. Stevens, L.M., et al., *Recommendations for Reporting Machine Learning Analyses in Clinical Research.* Circ Cardiovasc Qual Outcomes, 2020. **13**(10): p. e006556.

26. Wolff, R.F., et al., *PROBAST: A Tool to Assess the Risk of Bias and Applicability of Prediction Model Studies.* Ann Intern Med, 2019. **170**(1): p. 51-58.

27. Lambin, P., et al., *Radiomics: the bridge between medical imaging and personalized medicine.* Nat Rev Clin Oncol, 2017. **14**(12): p. 749-762.

28. Luo, W., et al., *Guidelines for Developing and Reporting Machine Learning Predictive Models in Biomedical Research: A Multidisciplinary View.* J Med Internet Res, 2016. **18**(12): p. e323.

29. Moons, K.G., et al., *Transparent Reporting of a multivariable prediction model for Individual Prognosis or Diagnosis (TRIPOD): explanation and elaboration.* Ann Intern Med, 2015. **162**(1): p. W1-73.

30. Wilkinson, M.D., et al., *The FAIR Guiding Principles for scientific data management and stewardship.* Sci Data, 2016. **3**: p. 160018.

31. van Royen, F.S., et al., *Five critical quality criteria for artificial intelligence-based prediction models.* Eur Heart J, 2023. **44**(46): p. 4831-4834.
