# Supplementary material for: Pitfalls in Developing Machine Learning Models for Predicting Cardiovascular Diseases: Challenge and Solutions
Source: J Med Internet Res. 2024 Jul 26;26:e47645. doi: 10.2196/47645 (PMC11316160; doi:10.2196/47645)
Supplement: Multimedia Appendix 3 [file jmir_v26i1e47645_app3.docx]

Supplementary material 3: The framework for pitfalls in developing ML models for predicting CVDs.

| **Evaluation Content (Grade Ⅰ)** | **Evaluation Content (Grade Ⅱ)** | **Evaluation Content (Grade Ⅲ)** | **Guideline No.^a^** | **ML for CVDs Prediction** | **Research References** | **Pitfalls** |
| --- | --- | --- | --- | --- | --- | --- |
| 1 Data quality | Data Acquisition | data/ training data/ baseline;  data collection, analysis, deployment, and use/ data collection methods;  data quality/ known quality issues with the data;  specifications, design, and the parameters;  data source/ provenance/ origin;  blinding;  measurement methods;  data harmonization;  definition and assessment/ predictors’ definition;  patients recruition;  eligibility criteria;  inclusions and exclusions;  reusability;  interoperability;  findability;  accessibility;  data availability;  link to data or data request mechanism;  input data type (structured or unstructured)/ continuous and categorical predictors;  image data processing  (data overlap  imaging protocol  definition of non-radiomic predictor variables  definition of the reference standard  metadata on images and potential biases  imaging modalities  confounding artifacts  define image datasets (training, validation, test)  relation of test dataset and proposed clinical setting  multiple segmentations  phantom study on all scanners  imaging at multiple time points);  clinical evaluation;  human–AI interaction;  data management/ data curation | 1, 2, 3, 4, 6, 7, 8, 10, 11, 12, 14, 15, 16, 17, 18, 19, 21, 23, 25, 26, 27, 28, 29, 30 | data source/ provenance/ origin;  data quality/ known quality issues with the data;  measurement methods;  data collection, analysis, deployment, and use/ data collection methods | [1]Han et al.  [2]Rodriguez et al.  [3]Teoh et al. | - 1. data recourse |
|  |  |  |  |  | [4]Benjamins et al.  [5]Liu et al.  [6]Tsoi et al. | 1.2 subjective factor of researchers |
|  |  |  |  |  | [6]Tsoi et al.  [7]Sung et al.  [8]Ayala Solares et al.  [9]Cho et al. | 1.4 parameter acquisition method |
|  | Data Processing | data abstraction, cleaning, preparation;  pre/post data processing/ data processing;  input data;  format/describe data;  data transformations;  normalize variables/ data standardization;  data characterization;  uncertainty measure;  data dimensionality reduction ;  data augmentation;  model pre-training;  statistical methods;  cut-off analyses;  missing data/ impute missing data/ handle missing value/ incomplete participants;  poor quality or unavailable input data;  remove outliers/handling outliers;  balance class;  describe feature selection | 2, 6, 7, 8, 10, 11, 15, 16, 17, 19, 20, 22, 23, 25, 26, 27, 28, 29 | missing data/ impute missing data/ handle missing value/ incomplete participants;  poor quality or unavailable input data | [1]Han et al.  [10]Sajeev et al.  [11]Nakanishi et al.  [12]Weng et al.  [13]Wang et al.  [14]Alaa et al.  [15].Dimopoulos et al. | 1.3 incomplete data |
| 2 Dataset Characteristics | Sampling Characteristics | data splitting/ data partitioning (splitting);  study design;  samples and features/ population;  baseline;  cohort characteristics/ sample record and measurement characteristics/ baseline characteristics;  baseline demographic and clinical characteristics;  number of sites;  countries of origin;  clinical validation;  key study dates(timing);  study setting;  treatments received;  sampling and sample size/ sufficient data/ sample or cohort size/ sample size calculation/ adequate sample size;  number of participants with the outcome;  biases;  outcome imbalances;  selection bias;  representative | 1, 3, 6, 7, 8, 9, 11, 12, 13, 14, 15, 16, 17, 18, 19, 20, 25, 26, 28, 29, 31 | cohort characteristics/ sample record and measurement characteristics/ baseline characteristics;  sampling and sample size/ sufficient data/ sample or cohort size/ sample size calculation/ adequate sample size;  number of participants with the outcome | [16]Ponomartseva et al.  [17]Chan et al. | 2.1 small sample size |
|  |  |  |  |  | [1]Han et al.  [18]Jdanov et al.  [19]Goldstein et al. | 2.2 low event rate |
|  | Algorithm Selection | deine analysis goal;  identify ML task;  select AI or mathematical methods/ select the best model/ rationale for selecting the machine learning algorithm;  define use of simple / complex models;  benchmark complex models;  data split methodology/ data splitting;  redundancy between data splits;  data distribution;  characteristics relevant for detecting data shift and drift;  model development (unadjusted association between each candidate predictor and outcome) | 7, 8, 14, 16, 19, 22, 29 | select AI or mathematical methods/ select the best model/ rationale for selecting the machine learning algorithm;  data distribution | [13]Hanyin et al.  [20]Angeli et al.  [21]Lip et al.  [22]Aniruddha et al.  [23]Li et al.  [24]Zhuang et al.  [25]Bharath et al. | 2.3 characteristics of the data distribution |
|  | Multimodal Data-based Prediction Model | input data;  definition of non-radiomic predictor variables;  rationale for using a radiomic approach;  multivariable analysis with non radiomics features (for example, EGFR mutation);  detect and discuss biological correlates;  poor-quality data;  describe the AI system;  AI system outputs | 8, 11, 15, 27 | input data;  rationale for using a radiomic approach;  multivariable analysis with non radiomics features;  poor-quality data | [26]Pujadas et al.  [27]Lou et al.  [28]Chao et al.  [29]Salah et al. | 2.4 multimodal data-based prediction model |
| 3 Model Design and Statistical Methods | Outcome | outcome;  output;  outcome definition, timing, or determination;  objective, standard and prespecified;  definition of the reference standard;  definedor determined in a similar way/ definition;  time interval;  measure outcome reliably/ method of outcome; determination;  blinding;  exclusion of predictors | 8, 9, 11, 14, 20, 26, 29 | definition of the reference standard;  definedor determined in a similar way/ definition | [9]Chao et al.  [30]You et al. | 3.1 outcome definitions |
|  | Data Analysis and Model Optimization Methodology/ Methodological Conduct | datasets;  measure predictors at baseline;  data availability/ availability of configuration;  Intended use of results/ clear intended use;  feature statistics (e.g., reproducibility, feature selection)/ parameters;  avoid univariable selection;  data suitability;  segmentation reliability analysis;  feature scaling details (e.g., normalization, standardization);  addressing class imbalance/ class imbalance;  feature reduction or adjustment for multiple testing/ dimension reduction details;  fitting/ overfitting;  number of participants with the outcome;  regularization;  accounted for overfitting and optimism;  rigorous validation;  hyperparameter tuning method/ select; hyper-parameters/ final model hyperparameters;  model’s output adjustments;  Improved prediction;  model updating;  specific version of the AI model;  trigger situations;  randomized steps;  baseline model;  state-of-the-art approaches | 6, 7, 8, 9, 12, 13, 14, 15, 16, 24, 25, 26, 27, 29, 31 | avoid univariable selection;  fitting/ overfitting;  number of participants with the outcome;  regularization;  accounted for overfitting and optimism;  rigorous validation | [15]Ward et al.  [31]Chua et al.  [32]Chen et al.  [33]Suri et al.  [34]Kakadiaris et al. | 3.2incomplete inclusion of covariates |
|  |  |  |  |  | [12]Weng et al.  [35]Commandeur et al.  [36]Voss et al. | 3.3overfitting |
|  | Model Performance | AI model building methodology;  evaluation measures, training protocols, and validation;  validation method and performance metrics;  testing technique (e.g., internal, external);  training;  validation;  internal validation results;  model evaluation/ model performance evaluation/ evaluation/ evaluation method;  evaluation of calibration and discrimination/  discrimination statistics and calibration statistics;  model evaluation on an external dataset;  availability of evaluation;  Test metrics;  model performance/ product performance metrics/ performance metrics and rationale for choosing/ performance measures/ algorithm performance evaluation metrics/ report the final model and performance;  uncertainty evaluation and measures (e.g., confidence intervals)/ confidence;  statistical performance comparison (e.g., DeLong's test)/ comparison;  appropriate statistical significance;  coverage and edge cases;  result analysis;  stability and Sensitivity Analysis;  critical analysis;  error analysis/ performance errors;  bias assessment;  benchmarking, technical comparison, and novelty;  existing model performance benchmarks;  justify the best-performing model;  compared to the current best technology gain in statistical performance;  comparison to ‘gold standard’ | 1, 5, 6, 7, 8, 10, 14, 15, 16, 17, 18, 19, 22, 21, 24, 25, 26, 27, 28, 29 | evaluation measures, training protocols, and validation;  model evaluation/ model performance evaluation/ evaluation/ evaluation method;  evaluation of calibration and discrimination/  discrimination statistics and calibration statistics;  model evaluation on an external dataset;  availability of evaluation;  test metrics;  model performance/ product performance metrics/ performance metrics and rationale for choosing/ performance measures/ algorithm performance evaluation metrics/ report the final model and performance | [23]Li et al.  [37]Polonsky et al. | 3.4 evaluation criteria defections |
| 4 Clinic Implication | Model Implementation/ Application and Replicability/ Reproducibility | strengths and limitations (e.g., bias and generalizability issues);  robustness/ adversarial robustness;  rigorous validation;  report the final model;  generalization performance/ generalisability;  target condition applicability;  clinical utility assessment;  cost or consequence of errors/ implications of errors; made by the model on clinical and economic outcomes;  resourcing;  cost and resource impact;  practical implications;  potential clinical utility;  users recruition;  familiarize the users with the AI system;  present clinical acceptability and user perceptions;  model and processing description;  Pre-trained model;  hyperparameter tuning;  re-running;  model specification;  execution environment;  transparency/ organisational transparency;  publicly evaluate algorithm;  open Science/ open science and data;  AI model/data accessible;  reproducibility and code reuse;  reporting and reproducibility;  transferable;  source code/ consider sharing code or scripts on public repositories;  availability of software;  openness of data and software;  code, software, and all other relevant parts accessible;  provide a data dictionary;  document detailing software and libraries;  clear and accessible data usage license;  domain-relevant community standards;  standardized communications protocol;  clinical implication | 3, 4, 5, 6, 7, 8, 10, 11, 12, 14, 15, 16, 18, 19, 20, 21, 22, 24, 25, 27, 28, 30, 31 | strengths and limitations (e.g., bias and generalizability issues);  robustness/ adversarial robustness;  rigorous validation;  generalization performance/ generalisability;  target condition applicability | [15]Ward et al.  [38]Bouzid et al.  [39]Unnikrishnan et al.  [40]Zarkogianni et al. | 4.1 problem of generalization |
|  | Model Explainability | algorithm development;  model complexity;  define use of simple/ complex models;  account for complexities in the data;  uncertainty;  transparency;  explainability/ interpretable/ interpretability/ interpretation/ intelligible/ interpreting the modela;  explainability methods/ model explanation/ interpretability and explainability methods;  intelligibility of output;  documentation and annotations;  human–ai interaction;  feature importance analysis;  lime (local interpretable model-agnostic explanations);  counterfactual analysis;  model distillation;  shapley additive explanations;  attention visualization;  plausibility of model outputs/ results justified;  performance metrics and data partitions(results);  results;  interpretation of model's results by an end-user;  user feedback | 3, 4, 5, 6, 7, 8, 10, 13, 14, 15, 17, 18, 19, 20, 21, 22, 26, 28, 29 | algorithm development;  model complexity;  define use of simple/ complex models;  account for complexities in the data;  explainability/ interpretable/ interpretability/ interpretation/ intelligible/ interpreting the modela;  explainability methods/ model explanation/ interpretability and explainability methods;  human–AI interaction | [1]Han et al.  [12]Weng et al.  [29]Salah Al-Zaiti et al.  [41]Xuan et al.  [42]Wang et al. | 4.2 lack of interpretability |
|  | AI Ethics | AI-ethics/ ethics;  ethical guidelines;  human-in-the-loop evaluation;  technical task-specific evaluation;  ethical data sourcing;  data annotation (ground truth)/ ground truth;  identification of errors;  diagnosis;  demographic disparities;  real world;  inclusivity and diversity;  ethical considerations and methodological biases;  data governance;  responsibility/ accountability  (model performance monitoring;  electronic monitoring;  prioritization of results;  bias detection and mitigation;  data bias analysis;  bias assessment;  applicability and bias;  bias mitigation;  explainability and interpretability;  transparency and auditing;  transparency;  ethical and other regulatory approvals obtained;  error analysis and feedback mechanisms;  failure modes and risk analysis);  assurance of patient safety/ patient safety/ safety and errors;  assessing security vulnerabilities;  safety constraints and compliance;  user safety measures;  safety and privacy;  data privacy compliance;  beneficence;  support for intended use;  potential impacts on the healthcare team and patients;  fair commercialisation | 2, 3, 4, 5, 6, 7, 10, 11, 12, 13, 15, 18, 21 | AI-ethics/ ethics;  demographic disparities;  real world;  inclusivity and diversity;  ethical considerations and methodological biases | [12]Weng et al.  [43]Kim et al.  [44]Segar et al. | 4.3 AI-ethics |

**^a^Guideline No.:** Number of guideline shown in Multimedia Appendix 2

**References**

1. Han, D., et al., *Machine learning based risk prediction model for asymptomatic individuals who underwent coronary artery calcium score: Comparison with traditional risk prediction approaches.* J Cardiovasc Comput Tomogr, 2020. **14**(2): p. 168-176.

2. Rodriguez, F., et al., *Atherosclerotic Cardiovascular Disease Risk Prediction in Disaggregated Asian and Hispanic Subgroups Using Electronic Health Records.* J Am Heart Assoc, 2019. **8**(14): p. e011874.

3. Teoh, D., *Towards stroke prediction using electronic health records.* BMC Med Inform Decis Mak, 2018. **18**(1): p. 127.

4. Benjamins, J.W., et al., *Enhancing cardiovascular artificial intelligence (AI) research in the Netherlands: CVON-AI consortium.* Neth Heart J, 2019. **27**(9): p. 414-425.

5. Liu, L., et al., *An early aortic dissection screening model and applied research based on ensemble learning.* Ann Transl Med, 2020. **8**(23): p. 1578.

6. Tsoi, K.K.F., et al., *Machine Learning Clustering for Blood Pressure Variability Applied to Systolic Blood Pressure Intervention Trial (SPRINT) and the Hong Kong Community Cohort.* Hypertension, 2020. **76**(2): p. 569-576.

7. Sung, J.M., et al., *Development and verification of prediction models for preventing cardiovascular diseases.* PLoS One, 2019. **14**(9): p. e0222809.

8. Ayala Solares, J.R., et al., *Long-Term Exposure to Elevated Systolic Blood Pressure in Predicting Incident Cardiovascular Disease: Evidence From Large-Scale Routine Electronic Health Records.* J Am Heart Assoc, 2019. **8**(12): p. e012129.

9. Cho, I.J., et al., *Development and External Validation of a Deep Learning Algorithm for Prognostication of Cardiovascular Outcomes.* Korean Circ J, 2020. **50**(1): p. 72-84.

10. Sajeev, S., et al., *Predicting Australian Adults at High Risk of Cardiovascular Disease Mortality Using Standard Risk Factors and Machine Learning.* Int J Environ Res Public Health, 2021. **18**(6).

11. Nakanishi, R., et al., *Machine Learning Adds to Clinical and CAC Assessments in Predicting 10-Year CHD and CVD Deaths.* JACC Cardiovasc Imaging, 2021. **14**(3): p. 615-625.

12. Weng, S.F., et al., *Can machine-learning improve cardiovascular risk prediction using routine clinical data?* PLoS One, 2017. **12**(4): p. e0174944.

13. Wang, H., et al., *Using Machine Learning to Integrate Socio-Behavioral Factors in Predicting Cardiovascular-Related Mortality Risk.* Stud Health Technol Inform, 2019. **264**: p. 433-437.

14. Alaa, A.M., et al., *Cardiovascular disease risk prediction using automated machine learning: A prospective study of 423,604 UK Biobank participants.* PLoS One, 2019. **14**(5): p. e0213653.

15. Ward, A., et al., *Machine learning and atherosclerotic cardiovascular disease risk prediction in a multi-ethnic population.* NPJ Digit Med, 2020. **3**: p. 125.

16. Ponomartseva, D.A., et al., *Prediction model for thyrotoxic atrial fibrillation: a retrospective study.* BMC Endocr Disord, 2021. **21**(1): p. 150.

17. Chan, K.L., et al., *Early Identification of High-Risk TIA or Minor Stroke Using Artificial Neural Network.* Front Neurol, 2019. **10**: p. 171.

18. Jdanov, D.A., et al., *Recalibration of the SCORE risk chart for the Russian population.* Eur J Epidemiol, 2014. **29**(9): p. 621-8.

19. Goldstein, B.A., et al., *Near-term prediction of sudden cardiac death in older hemodialysis patients using electronic health records.* Clin J Am Soc Nephrol, 2014. **9**(1): p. 82-91.

20. Angeli, F., G. Reboldi, and P. Verdecchia, *Hypertension and the J-curve phenomenon: implications for tight blood pressure control.* Hypertension Research, 2012. **36**(2): p. 109-111.

21. Lip, S., et al., *Diastolic Blood Pressure J-Curve Phenomenon in a Tertiary-Care Hypertension Clinic.* Hypertension, 2019. **74**(4): p. 767-775.

22. Dutta, A., et al., *An efficient convolutional neural network for coronary heart disease prediction.* Expert Systems with Applications, 2020. **159**.

23. Li, Y., et al., *Consistency of variety of machine learning and statistical models in predicting clinical risks of individual patients: longitudinal cohort study using cardiovascular disease as exemplar.* BMJ, 2020. **371**: p. m3919.

24. Zhuang, X.D., et al., *Deep Phenotyping and Prediction of Long-term Cardiovascular Disease: Optimized by Machine Learning.* Can J Cardiol, 2022. **38**(6): p. 774-782.

25. Ambale-Venkatesh, B., et al., *Cardiovascular Event Prediction by Machine Learning: The Multi-Ethnic Study of Atherosclerosis.* Circ Res, 2017. **121**(9): p. 1092-1101.

26. Pujadas, E.R., et al., *Prediction of incident cardiovascular events using machine learning and CMR radiomics.* Eur Radiol, 2023. **33**(5): p. 3488-3500.

27. Lou, Y.S., et al., *Extensive deep learning model to enhance electrocardiogram application via latent cardiovascular feature extraction from identity identification.* Comput Methods Programs Biomed, 2023. **231**: p. 107359.

28. Chao, H., et al., *Deep learning predicts cardiovascular disease risks from lung cancer screening low dose computed tomography.* Nat Commun, 2021. **12**(1): p. 2963.

29. Al-Zaiti, S., et al., *Machine learning-based prediction of acute coronary syndrome using only the pre-hospital 12-lead electrocardiogram.* Nat Commun, 2020. **11**(1): p. 3966.

30. You, J., et al., *Development of machine learning-based models to predict 10-year risk of cardiovascular disease: a prospective cohort study.* Stroke Vasc Neurol, 2023.

31. Chua, W., et al., *Data-driven discovery and validation of circulating blood-based biomarkers associated with prevalent atrial fibrillation.* Eur Heart J, 2019. **40**(16): p. 1268-1276.

32. Chen, X., et al., *Joint effect of elevated-c-reactive protein level and hypertension on new-onset stroke: A nationwide prospective cohort study of CHARLS.* Front Public Health, 2022. **10**: p. 919506.

33. Suri, J.S., et al., *Understanding the bias in machine learning systems for cardiovascular disease risk assessment: The first of its kind review.* Comput Biol Med, 2022. **142**: p. 105204.

34. Kakadiaris, I.A., et al., *Machine Learning Outperforms ACC / AHA CVD Risk Calculator in MESA.* J Am Heart Assoc, 2018. **7**(22): p. e009476.

35. Commandeur, F., et al., *Machine learning to predict the long-term risk of myocardial infarction and cardiac death based on clinical risk, coronary calcium, and epicardial adipose tissue: a prospective study.* Cardiovasc Res, 2020. **116**(14): p. 2216-2225.

36. Voss, R., et al., *Prediction of risk of coronary events in middle-aged men in the Prospective Cardiovascular Munster Study (PROCAM) using neural networks.* Int J Epidemiol, 2002. **31**(6): p. 1253-62; discussion 1262-64.

37. Polonsky, T.S., et al., *Coronary artery calcium score and risk classification for coronary heart disease prediction.* JAMA, 2010. **303**(16): p. 1610-6.

38. Bouzid, Z., et al., *In Search of an Optimal Subset of ECG Features to Augment the Diagnosis of Acute Coronary Syndrome at the Emergency Department.* J Am Heart Assoc, 2021. **10**(3): p. e017871.

39. Unnikrishnan, P., et al., *Development of Health Parameter Model for Risk Prediction of CVD Using SVM.* Comput Math Methods Med, 2016. **2016**: p. 3016245.

40. Zarkogianni, K., M. Athanasiou, and A.C. Thanopoulou, *Comparison of Machine Learning Approaches Toward Assessing the Risk of Developing Cardiovascular Disease as a Long-Term Diabetes Complication.* IEEE J Biomed Health Inform, 2018. **22**(5): p. 1637-1647.

41. Xuan, X., et al., *VAC-CNN: A Visual Analytics System for Comparative Studies of Deep Convolutional Neural Networks.* IEEE Trans Vis Comput Graph, 2022. **28**(6): p. 2326-2337.

42. Wang, Z.J., et al., *CNN Explainer: Learning Convolutional Neural Networks with Interactive Visualization.* IEEE Trans Vis Comput Graph, 2021. **27**(2): p. 1396-1406.

43. Kim, J., U. Kang, and Y. Lee, *Statistics and Deep Belief Network-Based Cardiovascular Risk Prediction.* Healthc Inform Res, 2017. **23**(3): p. 169-175.

44. Segar, M.W., et al., *Development and Validation of Machine Learning-Based Race-Specific Models to Predict 10-Year Risk of Heart Failure: A Multicohort Analysis.* Circulation, 2021. **143**(24): p. 2370-2383.
